# Oral cysteamine as an adjunct treatment in cystic fibrosis pulmonary exacerbations: An exploratory randomized clinical trial

**DOI:** 10.1371/journal.pone.0242945

**Published:** 2020-12-28

**Authors:** Graham Devereux, Danielle Wrolstad, Stephen J. Bourke, Cori L. Daines, Simon Doe, Ryan Dougherty, Rose Franco, Alastair Innes, Benjamin T. Kopp, Jorge Lascano, Daniel Layish, Gordon MacGregor, Lorna Murray, Daniel Peckham, Vincenzina Lucidi, Emma Lovie, Jennifer Robertson, Douglas J. Fraser-Pitt, Deborah A. O'Neil

**Affiliations:** 1 Liverpool School of Tropical Medicine, Liverpool, United Kingdom; 2 Precision for Medicine, Oncology and Rare Disease, Carlsbad, CA, United States of America; 3 Royal Victoria Infirmary, Newcastle, United Kingdom; 4 Banner University of Arizona Medical Center, Tucson, Arizona, United States of America; 5 San Francisco Critical Care Medical Group California Pacific Medical Center, San Francisco, United States of America; 6 The Medical College of Wisconsin/Froedtert Hospital, Milwaukee, Wisconsin, United States of America; 7 Western General Hospital, Edinburgh, United Kingdom; 8 Nationwide Children's Hospital, Columbus, OH, United States of America; 9 University of Florida, Gainesville, Florida, United States of America; 10 Central Florida Pulmonary Group, Orlando, Florida, United States of America; 11 Queen Elizabeth University Hospital, Glasgow, United Kingdom; 12 Raigmore Hospital, Inverness, United Kingdom; 13 St James’s University Hospital, Leeds, United Kingdom; 14 Ospedale Padiatrico Bambino Gesu Centro Fibrosi Cistica, Rome, Italy; 15 NovaBiotics Ltd, Aberdeen, United Kingdom; National Institute for Infectious Diseases (L. Spallanzani), ITALY

## Abstract

**Background:**

Emerging data suggests a possible role for cysteamine as an adjunct treatment for pulmonary exacerbations of cystic fibrosis (CF) that continue to be a major clinical challenge. There are no studies investigating the use of cysteamine in pulmonary exacerbations of CF. This exploratory randomized clinical trial was conducted to answer the question: In future pivotal trials of cysteamine as an adjunct treatment in pulmonary exacerbations of CF, which candidate cysteamine dosing regimens should be tested and which are the most appropriate, clinically meaningful outcome measures to employ as endpoints?

**Methods and findings:**

Multicentre double-blind randomized clinical trial. Adults experiencing a pulmonary exacerbation of CF being treated with standard care that included aminoglycoside therapy were randomized equally to a concomitant 14-day course of placebo, or one of 5 dosing regimens of cysteamine. Outcomes were recorded on days 0, 7, 14 and 21 and included sputum bacterial load and the patient reported outcome measures (PROMs): Chronic Respiratory Infection Symptom Score (CRISS), the Cystic Fibrosis Questionnaire–Revised (CFQ-R); FEV_1_, blood leukocyte count, and inflammatory markers. Eighty nine participants in fifteen US and EU centres were randomized, 78 completed the 14-day treatment period. Cysteamine had no significant effect on sputum bacterial load, however technical difficulties limited interpretation. The most consistent findings were for cysteamine 450mg twice daily that had effects additional to that observed with placebo, with improved symptoms, CRISS additional 9.85 points (95% CI 0.02, 19.7) p = 0.05, reduced blood leukocyte count by 2.46x10^9^ /l (95% CI 0.11, 4.80), p = 0.041 and reduced CRP by geometric mean 2.57 nmol/l (95% CI 0.15, 0.99), p = 0.049.

**Conclusion:**

In this exploratory study cysteamine appeared to be safe and well-tolerated. Future pivotal trials investigating the utility of cysteamine in pulmonary exacerbations of CF need to include the cysteamine 450mg doses and CRISS and blood leukocyte count as outcome measures.

**Clinical trial registration:**

NCT03000348; www.clinicaltrials.gov.

## Introduction

Cystic fibrosis (CF) continues to be a life-limiting autosomal recessive disease. Although high quality multidisciplinary care has increased median predicted survival to 47 years of age, median age of death is currently around thirty years [1]. The major cause of morbidity and mortality in CF is progressive lung disease due to pulmonary infection and inflammation [2].

Despite recent interventions to reduce pulmonary exacerbation frequency (e.g. CFTR modulator therapies) they continue to be common, with one in three patients requiring at least one annual course of intravenous antibiotics [3]. Treatment failure rate is considerable with lung function failing to return to 90% of baseline in 15–25% of episodes [4,5]. Pulmonary exacerbations adversely impact quality of life, incur significant healthcare costs, and are associated with a more rapid decline in lung function [6,7]. To mitigate these inevitable consequences, new and better exacerbation-specific interventions are required.

Cysteamine (HSCH_2_CH_2_NH_2_) is an aminothiol product of coenzyme A metabolism that has been licensed for over 20 years for the treatment of cystinosis [8,9]. *In vitro* and Phase 1/2a studies have demonstrated that cysteamine has multiple properties potentially beneficial as an adjunct treatment in pulmonary exacerbations of CF. The drug exhibits antimicrobial, (anti-biofilm, antibiotic-potentiating, anti-virulence), anti-inflammatory and mucoactive properties [10–13]. Cysteamine is also a regulator of proteostasis and has been shown to stimulate autophagy in cells with the ΔF508 mutation, helping to stabilise CFTR at the plasma membrane [14]. It has been used alone, and in combination with epigallocatechin gallate (EGCG) enhancing autophagy in mouse models and improving CFTR function in primary cells [15]. Cysteamine also potentiates autophagy in macrophages with ΔF508, improving the elimination of engulfed bacterial pathogens [16,17]. A phase 1/2a trial demonstrated that oral cysteamine is absorbed and accumulates in bronchial secretions in people with CF, but drug efficacy in acute exacerbations has not yet been investigated [18].

The objectives of this exploratory trial were to identify candidate dosing regimens of oral cysteamine, and patient reported outcome measures (PROM) to include in future pivotal studies of oral cysteamine as an adjunct intervention in pulmonary exacerbations of CF.

## Methods

### Trial design

This was a parallel-group, randomized placebo controlled trial with a 1:1:1:1:1:1 allocation ratio comparing the addition of 5 differing cysteamine dosing regimens or placebo to the standard treatment of adults with CF experiencing a pulmonary exacerbation.

### Participants, eligibility criteria, and settings

Participants were recruited from 15 CF centres in the UK, EU and USA between 12th January 2017 and 21^st^ March 2018. Participants were aged ≥18 years with an established diagnosis of CF lung disease, chronic infection with Gram-negative organisms, experiencing a new CF pulmonary exacerbation requiring treatment that included an aminoglycoside antibiotic, weighed >40kg, and had an FEV_1_ >30% predicted in the previous 6 months. The diagnosis of CF lung disease with Gram-negative infection was established from clinical records. Exacerbations were confirmed by ≥4 defining symptoms as described by Fuchs [19]. Exclusion criteria included hypersensitivity to cysteamine, excipients or penicillamine, and transplant recipients. Participants were recruited by clinic staff and the treatment setting for pulmonary exacerbation was as per local practice for each centre (a mix of inpatient and community therapy).

The trial sponsor was NovaBiotics and the trial was approved by each site’s Institutional Review Board. Trial registrations FDA IND 127409, EudraCT 2015-004986-99, www.clinicaltrials.gov NCT03000348 (registered 22nd December 2016). All participants provided written informed consent.

### Interventions

**Fig 1:**
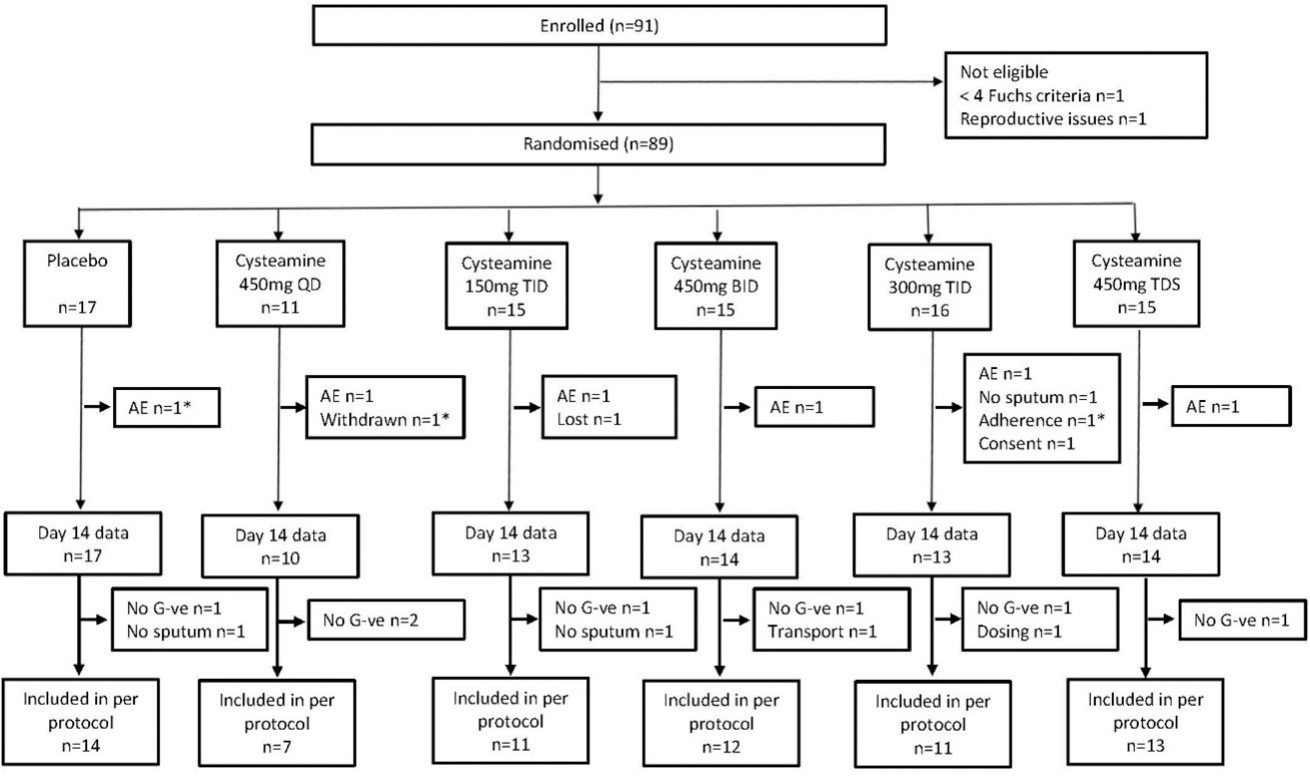
Diagram illustrating enrolment, randomisation and follow up of participants. * Data available at day 14. AE: Adverse event, QD: once a day, BID: twice a day, TID: three times a day. No G-ve: No Gram negative organism isolated from baseline sputum sample.

Cysteamine (as mercaptamine bitartrate) in 150mg hard gel capsules for oral administration was supplied by Recordati Industria Chimica e Farmaceutica S.p.A. Milan, Italy. Placebo comprised the excipients in identical capsules and in order to maintain double-blinding, were packaged in ‘smell-masked’ blister packs to mirror the odour of the treatment capsules. Participants were randomly assigned to one of six treatment groups in equal ratio: placebo, cysteamine 150mg three times daily, cysteamine 450mg once daily, cysteamine 300mg three times daily, cysteamine 450mg twice daily and cysteamine 450mg three times daily. Dosing schedules were based on those for cystinosis and the findings of a previous trial [18] and are outlined in Fig 1. Cysteamine doses were either administered in oral 450mg boluses or an equivalent total oral daily dose three times a day, to investigate the relative contributions of peak concentrations and total daily dose to any therapeutic effect, i.e. 450mg once daily, 150mg three times daily, and 450mg twice daily, 300mg three times daily. The treatment period with antibiotics was 14 days and each participant took 3 study capsules (non-cysteamine capsules made up with placebo), 3 times a day for 14 days.

### Outcomes

Outcome data were collected by face-to-face assessments at recruitment/baseline (day 0), 7, 14 and 21 days. Participants ceasing trial medication were encouraged to attend remaining scheduled assessments.

Sputum samples were obtained at each assessment and the following sputum based outcomes were quantified in central laboratories: a). Gram-negative bacterial load expressed as colony-forming units (CFU) per ml, b). sputum interleukin (IL)-8, c). sputum neutrophil elastase (NE), and d) sputum cysteamine (day 14 only). The PROMs administered at each assessment: Cystic Fibrosis Respiratory Symptom Domain-Chronic Respiratory Infection Symptom Score (CFRSD-CRISS) [20,21], the Cystic Fibrosis Questionnaire–Revised (CFQ-R) [22], and the Jarad & Sequeiros Symptom Score (JSSS) [23]. Venous blood samples were obtained at each assessment and the following outcomes were quantified in central laboratories: a). haematology including leukocyte count b). biochemistry, c). C-reactive protein (CRP), and d) blood cysteamine (day 14 only). Additional outcomes collected at each assessment visit were FEV_1_ percent predicted, weight, routine urinalysis, adverse events (AE)/reactions, serious adverse events (SAE)/reactions, and adherence.

### Sample size/power considerations

The sample size of approximately 120 patients with pulmonary exacerbations of CF with 20 patients in each group was selected empirically without a formal statistical assumption. The sample size selection was considered to be appropriate for an exploratory study to determine the optimal dose and regimen based on evidence of efficacy and acceptable safety and tolerability profile as well as establish point estimates and variability for efficacy endpoints for future evaluation. At the time the study was being designed there was a lack of published PROM data from observational studies and interventional trials of exacerbations of CF. With a sample size of 20 patients in each group the study had 80% power to detect a 1.2 log reduction over placebo of sputum Gram-negative bacterial load, assuming a 5% withdrawal rate, a standard deviation of 1.31, based on a two-sided, two-sample t-test at the 5% level of significance [24]. This estimated standard deviation is that reported for a 2-week study of CF patients with Pseudomonas aeruginosa who were treated during exacerbations with 2 weeks of intravenous tobramycin [25].

### Interim analyses and stopping guidelines

Not applicable.

### Randomization

Randomization in 1:1:1:1:1:1 allocation to the six test groups was achieved via a web-based computer-generated program, verified for accuracy using strict quality control procedures. Randomization was centralized and each site was assigned blocks of six treatments in the randomization scheme.

### Blinding

Participants and trial staff were blinded to study treatment allocation.

### Statistical analysis

All analyses were governed by a Statistical Analysis Plan. The intention to treat (ITT) analysis included all participants who had taken at least one dose of trial drug. A per-protocol analysis performed as a sensitivity analysis comprised all participants whose baseline sputum cultured Gram-negative organisms and who completed the 14-day treatment period without protocol violations.

The primary outcome of change in sputum Gram-negative bacterial load from Baseline (Day 0) to Day 14 was compared between randomized groups using all available data without imputation in a linear mixed model for repeated measures (MMRM) with an unstructured covariance matrix, factors for treatment group (6 levels: placebo, 450mg once daily (QD), 150mg three times daily (TID), 450mg twice daily (BID), 300mg TID, and 450TID), assessment (2 levels: Day 7 and 14), and assessment by treatment group interaction and the baseline value as a continuous covariate. Exploratory ANCOVA modelling was performed to assess the influence of select baseline factors on the change from baseline at day 14. We did not adjust for centre as an effect because 11 of the 15 sites recruited less than 10 participants each such that there were often only one or two participants within each treatment group at each site. With such low numbers within each treatment group at each site we anticipated that inclusion of ‘centre’ would result in convergence issues as well as complexities with interpretability and exploratory analyses confirmed that this was indeed the case. A 5% two-sided significance level was used throughout and no adjustment for multiple comparisons was performed because of the exploratory nature of the study. Secondary outcomes were similarly analysed. Examination of the residuals (eg, Q-Q and density plots, residual plots, variance of residuals within groups, sensitivity to outliers) for the MMRM and ANCOVA models confirmed that the necessary normality assumptions were not contravened. Analyses were performed using Base SAS®, v9.4. SAS Institute Inc.

## Results

Participant involvement in the trial is outlined in Fig 1. Ninety-one participants were enrolled with 89 being randomized and commenced on trial medication. Seventy-eight participants completed the 14-day treatment period (study drug and antibiotics), three who discontinued the trial drug attended Day 14 assessments.

Discontinuation of trial drug was lowest in the placebo group (6%) and highest in the 300mg TID group (25%). Reasons for discontinuation included AE (n = 6), loss to follow-up (n = 1), non-adherence (n = 1), physician decision (n = 1), consent withdrawn (n = 1), and failure to expectorate sputum (n = 1).

Sixty-eight participants were included in the per-protocol analysis. The reasons for exclusion from the per-protocol analysis in addition to not completing the 14-day treatment period were: no Gram-negative organisms isolated from baseline sputum (n = 7), inability to provide sputum sample(s) (n = 2), delayed transportation of sputum sample (n = 1), and inadvertent under dosing (n = 1). The decision to discontinue recruitment at about 90 participants, (primarily because of below target rate of recruitment) was made by the Data and Safety Monitoring Board (DSMB) and sponsor based on aggregated recruitment data.

Baseline characteristics of the participants are outlined in Table 1. The groups appeared to be balanced for age, BMI, FEV_1_, Fuchs criteria and sex. All participants were commenced on aminoglycoside antibiotics to treat the exacerbation (inclusion criterion). The antibiotics used to treat the exacerbations were overall balanced between groups, except for of beta-lactams in the placebo and 150mg TID groups. The groups were less balanced for the use of chronic therapies: pancreatic enzyme replacement therapy (PERT) 73–100%, mucolytics 67–93%, macrolides 47–80%, inhaled aminoglycosides 27–71%, inhaled colomycin 33–63% and ivacaftor or lumacaftor/ivacaftor 0–41%.

**Table 1:** Baseline characteristics and antibiotic treatment of participants allocated to trial treatment groups.

	Placebo n = 17	Cysteamine dose				
		450mg QD n = 11	150mg TID n = 15	450mg BID n = 15	300mg TID n = 16	450mg TID n = 15
Age (years), mean (SD)	27.2 (5.64)	27.5 (6.77)	32.5 (12.7)	32.3 (9.78)	31.4 (12.0)	27.5 (7.89)
Female n(%)	8 (47.1%)	6 (54.5%)	5 (33.3%)	8 (53.3%)	8 (50.0%)	8 (53.3%)
BMI (kg.m^2^) mean (SD)	20.2 (2.23)	20.3 (3.03)	20.7 (2.41)	21.5 (2.21)	20.5 (3.03)	21.7 (2.84)
FEV_1_% predicted mean (SD)	41.5 (15.3)	39.4 (19.8)	48.0 (18.3)	46.1 (22.7)	37.7 (13.4)	46.9 (20.6)
Fuchs’ criteria median (IQR)	5 (4, 6)	6 (5,6)	5 (4, 6)	5 (4, 7)	6 (4, 7)	6 (5, 8)
Enzyme replacement therapy n (%)	16 (94.1%)	8 (72.7%)	14 (93.3%)	15 (100%)	13 (81.3%)	14 (93.3%)
Mucolytics n (%)	12 (70.6%)	10 (90.9%)	10 (66.7%)	14 (93.3%)	12 (75.0%)	13 (86.7%)
Macrolides n (%)	11 (64.7%)	8 (72.7%)	7 (46.7%)	12 (80.0%)	8 (50.0%)	9 (60.0%)
Inhaled aminoglycosides n (%)	12 (70.6%)	3 (27.3%)	8 (53.3%)	8 (53.3%)	5 (31.3%)	6 (40.0%)
Inhaled colomycin n (%)	6 (35.3%)	5 (45.5%)	5 (33.3%)	6 (40.0%)	10 (62.5%)	5 (33.3%)
lumacaftor ± ivacaftor n(%)	7 (41.2%)	1 (9.1%)	3 (20.0%)	0	2 (12.5%)	5 (33.3%)
*Treatment of exacerbation*						
Aminoglycosides	17* (100%)	11 (100%)	15 (100%)	15 (100%)	16** (100%)	15 (100%)
Betalactams	12 (70.6%)	7 (63.6%)	15 (100%)	8 (53.3%)	9 (56.3%)	8 (53.3%)
Monobactam	4 (23.5%)	0 (0%)	5 (33.3%)	4 (26.7%)	5 (31.3%)	4 (26.7%)
Carbepenem	5 (29.4%)	3 (27.3%)	3 (20.0%)	5 (33.3%)	4 (25.0%)	4 (26.7%)
Glycopeptide	5 (29.4%)	3 (27.3%)	1 (6.7%)	0 (0%)	3 (18.8%)	1 (6.3%)

QD = once daily; BID = two times daily; TID = three times daily; TDD = total daily dose.

* one participant nebulised aminoglycoside

** two participants nebulised aminoglycosides.

### Sputum gram negative bacterial load

Table 2 outlines log_10_ transformed sputum Gram negative bacterial load. The mean (SD) changes from baseline to Day 14 were: -1.36 (2.27) for placebo, 0.12 (2.05) cysteamine 450mg QD, -1.24 (2.69) 150mg TID, -1.32 (2.30) 450mg BID, -0.98 (1.89) 300mg TID, 0.34 (2.27) 450mg TID. There were no statistically significant differences between any of the cysteamine treatment groups and placebo.

**Table 2:** Change from baseline to day 14 in Log_10_-transformed total gram-negative sputum bacterial load (CFU/mg).

	Placebo n = 17	Cysteamine dose				
		450mg QD n = 11	150mg TID n = 15	450mg BID n = 15	300mg TID n = 16	450mg TID n = 15
Baseline, CFU mean (SD)	6.67 (2.09)	4.76 (3.66)	6.43 (2.34)	7.08 (2.50)	7.21 (2.00)	5.89 (2.62)
Day 14 Change from Baseline Mean (SD)	-1.36 (2.27)	0.12 (2.05)	-1.24 (2.69)	-1.32 (2.30)	-0.98 (1.89)	0.34 (2.27)
Day 14 LSMD (cysteamine–placebo) mean (95% CI)		0.71 (-1.16, 2.58)	0.01 (-1.68, 1.71)	0.70 (-1.04, 2.45)	0.81 (-1.00, 2.62)	1.57 (-0.07, 3.20)
P		0.451	0.986	0.424	0.375	0.061

QD = once daily; BID = two times daily; TID = three times daily; TDD = total daily.

CFU = colony forming units; LSMD = least square mean difference.

Analysis using mixed model for repeated measures (MMRM).

### Patient reported outcome measures

Any effects of cysteamine were most evident at day 14 and not day 7. At day 14, the improvement in CFRSD-CRISS observed with cysteamine 450mg BID was greater than the improvement in CFRSD-CRISS with placebo by -9.85 points (95% CI -19.7, -0.02) p = 0.05 (Table 3). Analysis of the individual CFRSD-CRISS domains revealed that there were differences greater than that observed with placebo for: feeling feverish: [450mg QD mean -0.5 (95% CI -0.9, -0.1), p = 0.016; 450mg BID -0.4 (-0.7, 0.0) p = 0.043; 450mg TID -0.5 (-0.9, -0.1) p = 0.010], and chest tightness [450mg BID -0.6 (-1.2, 0.0) p = 0.038; 450mg TID -0.7 (-1.3, -0.1) p = 0.025]. Subgroup analyses for baseline medication use demonstrated effects on CFRSD-CRISS score at day 14 greater than those observed with placebo for participants using RhDNAse [450mg BID -14.2 (-24.7, -3.7), p = 0.009, 450mg TID -11.2, (-22.2, -0.2), p = 0.046] and for participants not using macrolides [450mg QD -19.3, (-36.7, -1.9), p = 0.030; 450mg BID -41.0 (-58.6, -23.6), p<0.0001; 450mg TID -17.8, (-33.3, -2.3), p = 0.025].

**Table 3:** Change from baseline in patient reported outcome measures.

	Placebo n = 17	Cysteamine dose				
		450mg QD n = 11	150mg TID n = 15	450mg BID n = 15	300mg TID n = 16	450mg TID n = 15
*CFRSD CRISS*						
Baseline, mean (SD)	48.5 (10.6)	48.9 (12.14)	47.5 (8.10)	54.3 (13.13)	51.0 (10.93)	56.1 (8.82)
Day 14 Change from Baseline Mean (SD)	-16.3 (15.0)	-24.3 (16.35)	-15.5 (12.48)	-28.1 (16.88)	-14.8 (8.53)	-23.9 (16.41)
Day 14 LSMD (cysteamine–placebo) mean (95% CI)		-7.36 (-18.2, 3.51)	0.57 (-9.47, 10.6)	-9.85 (-19.7, -0.02)	2.32 (-7.65, 12.3)	-6.27 (-16.1, 3.60)
P		0.181	0.910	0.050	0.644	0.210
*CFQ-R*						
Baseline, mean (SD)	47.1 (20.9)	45.5 (15.1)	44.8 (20.0)	38.2 (21.8)	36.8 (17.7)	31.9 (17.2)
Day 14 Change from Baseline Mean (SD)	29.9 (21.51)	24.4 (14.15)	19.7 (16.76)	31.3 (17.38)	21.8 (14.07)	28.6 (15.69)
Day 14 LSMD (cysteamine–placebo) mean (95% CI)		1.26 (-11.1, 13.6)	-3.09 (-14.5, 8.29)	5.20 (-5.96, 16.4)	-5.39 (-16.8, 6.00)	1.78 (-9.54, 13.1)
P		0.839	0.590	0.356	0.349	0.755
*Jarad & Sequeiros Symptom Score*						
Baseline, mean (SD)	10.6 (2.91)	9.8 (2.93)	10.1 (2.56)	11.3 (2.72)	11.1 (2.85)	12.3 (2.05)
Day 14 Change from Baseline Mean (SD)	-3.2 (2.90)	-2.9 (2.60)	-2.0 (2.48)	-4.3 (2.16)	-3.1 (2.63)	-4.2 (3.19)
Day 14 LSMD (cysteamine–placebo) mean (95% CI)		-0.08 (-1.80, 1.64)	0.73 (-0.86, 2.33)	-0.54 (-2.09, 1.01)	0.33 (-1.25, 1.91)	-0.03(-1.59, 1.54)
P		0.924	0.363	0.491	0.681	0.973

QD = once daily; BID = two times daily; TID = three times daily; TDD = total daily dose.

LSMD = least square mean difference.

Analysis using mixed model for repeated measures (MMRM).

Cysteamine had no significant effect on overall CFQ-R and JSSS scores, however at day 14 cysteamine 450mg BID had effects greater than those observed with placebo for the CFQ-R domains Health Perception 12.4 (95% CI, 0.34, 24.4), p = 0.044, and Vitality 14.6 (2.33, 26.8), p = 0.020.

### Other outcomes

Cysteamine 450mg BID had a greater effect than placebo in reducing day 14 blood leukocyte count by 2.46x10^9^ /l (95% CI 0.11, 4.80), p = 0.041 (Table 4). Cysteamine 450mg BID was the only dosing schedule that had a greater (but not statistically significant) increase in FEV_1_ relative to placebo at day 14 by 4.03% predicted (95% CI -3.12, 11.2) (Table 4). Cysteamine had no significant effects on sputum NE or IL-8 concentrations, but there was a significant difference in plasma CRP concentrations at 450mg BID compared with placebo with a LSMD of log_10_−0.41 nmol/l (95% CI -0.8243, -0.0020), p = 0.0489. CRP was also reduced across all cysteamine treatment groups compared with placebo using covariate adjusted analysis (p = 0.049).

**Table 4:** Change from baseline in white cell count, FEV_1_ and BMI.

	Placebo n = 17	Cysteamine dose				
		450mg QD n = 11	150mg TID n = 15	450mg BID n = 15	300mg TID n = 16	450mg TID n = 15
*White blood count x10*^*9*^*/l*						
Baseline, mean (SD)	12.42 (4.19)	10.72 (3.27)	12.14 (3.93)	10.69 (2.76)	10.76 (3.40)	13.54 (3.32)
Day 14 Change from Baseline Mean (SD)	-1.57 (4.72)	-2.22 (2.35)	-2.65 (3.91)	-3.42 (3.57)	-0.19 (3.84)	-4.07 (2.41)
Day 14 LSMD (cysteamine–placebo) mean (95% CI)		-1.32 (-3.90, 1.26)	-0.91 (-3.35, 1.52)	-2.46 (-4.80, -0.11)	0.37 (-2.10, 2.84)	-1.56 (-3.92, 0.81)
P		0.311	0.456	0.041	0.765	0.194
*FEV*_*1*_*% predicted*						
Baseline, mean (SD)	41.5 (15.31))	39.4 (19.81)	48.0 (18.26)	38.2 (21.8)	36.8 (17.7)	31.9 (17.2)
Day 14 Change from Baseline Mean (SD)	9.1 (14.03)	4.0 (5.14)	8.9 (10.87)	13.6 (10.83)	5.3 (6.65)	7.5 (7.07)
Day 14 LSMD (cysteamine–placebo) mean (95% CI)		-5.10 (-13.1, 2.87)	-0.15 (-7.53, 7.23)	4.03 (-3.12, 11.2)	-3.80 (-11.1, 3.49)	-2.27 (-9.43, 4.89)
P		0.207	0.967	0.356	0.303	0.529
*BMI kg/m*^*2*^						
Baseline, mean (SD)	20.15 (2.23)	20.30 (3.03)	20.65 (2.41)	21.46 (2.21)	20.47 (3.03)	21.67 (2.84)
Day 14 Change from Baseline Mean (SD)	0.34 (0.45)	0.64 (0.51)	0.34 (0.48)	0.37 (0.49)	0.23 (0.71)	0.32 (0.89)
Day 14 LSMD (cysteamine–placebo) mean (95% CI)		0.3 (-0.2, 0.8)	0.0 (-0.4, 0.5)	0.1 (-0.4, 0.5)	-0.1 (-0.6, 0.4)	0.1 (-0.4, 0.5)
P		0.208	0.975	0.752	0.702	0.911

QD = once daily; BID = two times daily; TID = three times daily; TDD = total daily dose.

LSMD = least square mean difference.

Analysis using mixed model for repeated measures (MMRM).

Further analyses adjusting for antibiotic regime used to treat the exacerbation or baseline use of mucolytics, inhaled antimicrobials, ivacaftor, lumacaftor/ivacaftor, FEV_1_, BMI, leukocyte count, or CFRSD-CRISS score did not substantially alter the observed effects. Additional analyses including 450mg QD, BID and TID in single models provided little evidence of linear or non-linear dose response effects. There were no significant differences at day 21 (**S1** Day 14 geometric mean (GM) (geometric SD, GSD) plasma concentrations were 45.3ng/ml (3.86) for the cysteamine 150mg TID regimen and 104ng/ml (4.55) for the cysteamine 300mg TID regimen. For the 450mg dose regimens the GM plasma concentrations were 85.7ng/ml (10.1) for 450mg QD, 129ng/ml (5.04) for 450mg BID, and 153ng/ml (3.06) for 450mg TID. The GM (GSD) sputum concentrations levels were 150ng/ml (1.00) and 284ng/ml (3.36) for the 150mg TID and 300mg TID dose regimens respectively. For the 450mg QD, BID, and TID dose regimens sputum concentrations were 342ng/ml (3.11), 234ng/ml (2.11), and 498ng/ml (2.75) respectively. For the participants in the placebo group, plasma and sputum cysteamine concentrations were less than the lower limits of quantification (20 and 300ng/ml respectively).

### Adherence and adverse events

Mean (SD) rates of adherence were 99% (3), 89% (28), 89% (29), 92% (20), 78% (39), and 94% (11), in placebo, cysteamine 450mg QD, 150mg TID, 450mg BID, 300mg TID, and 450mg TID groups, respectively.

No deaths occurred. There were 213 AEs (**S2 Table. Summary of participant reported adverse events (AEs) by study group**). All the cysteamine groups reported a higher incidence of AEs than the placebo group. Six SAEs were reported, balanced between the groups. One SAE was classed as a SUSAR in a participant in the 150mg TID group who developed depression. This subject experienced a similar episode several years prior. The five remaining SAEs comprised haemoptysis, axillary vein thrombosis, campylobacter sepsis, nephrolithiasis, and pulmonary exacerbation of CF. All SAEs resolved, and all, apart from the SUSAR, were considered unrelated to treatment. There were no clinically relevant differences in routine haematological indices. Two participants had mild increases in ALT/AST whilst taking cysteamine (to 90 U/l & 67 U/l), these started to improve while the participants were still taking trial drug.

## Discussion

This exploratory RCT was conducted to investigate the possible role of cysteamine as an adjunct treatment for pulmonary exacerbations of CF by identifying candidate dosing regimens and patient reported and laboratory-based outcome measures to include in future pivotal trials. The findings indicate that future studies should use 14-day courses of cysteamine and at least include the 450mg BID dosing regimen that after two weeks treatment improved symptoms (CFRSD-CRISS, p = 0.050), the CFQ-R domain scores of Vitality (p = 0.020) and Health Perception (p = 0.044), and reduced blood leukocyte count (p = 0.041) and CRP (p = 0.049). Symptom improvement was mostly related to the CFRSD-CRISS domains of feeling feverish (p = 0.043) and chest tightness (p = 0.038) and was evident in participants taking mucolytics/RhDNAse and most prominent in those not taking macrolides at baseline. The absence of any effects of the total daily dose being divided equally between three doses suggests that peak concentrations of cysteamine and not total daily dose are clinically important. The overall symptom impact identified with CFRSD-CRISS but not CFQ-R may reflect the CFRSD-CRISS focus on the previous 24 hours whereas the CFQ-R has a fourteen-day reference period. None of the cysteamine dosing regimens had a significant effect on sputum microbial load; however as discussed below, technical issues limit interpretation of these data. Cysteamine was reasonably well tolerated but as expected was associated with increased mild side effects typical of those reported in the cystinosis literature.

For CFRSD-CRISS a change of 16-points is the individual response criterion for standard of care treatment of pulmonary exacerbations and a change of 11 units is considered clinically significant [20,21,26]. In the present study the standard care/placebo group had a 16.3 point improvement in CFRSD-CRISS, whereas cysteamine 450mg BID resulted in a 28.1 point reduction in CFRSD-CRISS indicating, that cysteamine 450mg BID had an additional clinically significant effect over and above that observed with standard care. The 16.3 point improvement in CFRSD-CRISS observed in the current study for the placebo group is less than the 26.1 point reduction with standard care reported by the Standardized treatment of pulmonary exacerbations (STOP) study [27]. The most likely explanation for the greater improvement in symptom score with STOP is that 61% of STOP participants aged ≥18 years had >14 days of antibiotic therapy for their exacerbations, this contrasts with the 14 days of antibiotics received by the participants in the current study. The 26.1 point reduction in CFRSD-CRISS with STOP is comparable with the 28.1, 24.3 and 23.9 point reductions observed with 14 day courses of 450mg BID, QD and TID doses of cysteamine, respectively, suggesting, perhaps that the addition of oral cysteamine has the potential to shorten the duration of antibiotic therapy for pulmonary exacerbations of CF [27]. The 9.1% increase in FEV_1_ observed in the standard care/placebo group in the present study is comparable to the 9% improvement reported in STOP [27]. In the present study the participants allocated to 450mg BID cysteamine had a 13.6% increase in FEV_1_, however this was not statistically superior to standard care/placebo. The disparity between CFRSD-CRISS and FEV_1_ for the 450mg BID dose of cysteamine is consistent with the finding that CFRSD-CRISS is more sensitive than FEV_1_ in quantifying responses to treatment of pulmonary exacerbations of CF [21].

Cysteamine has mucolytic activity in *ex vivo* sputum as well as manifold anti-infective properties which can target both viruses and bacteria, key triggers of pulmonary exacerbations [10–13,28]. *In vitro* studies indicate that cysteamine may also have indirect antimicrobial properties through effects on the host. This includes increasing clearance of antibiotic-resistant pathogens from macrophages due to the potentiation of autophagy [17], and similarly may even restore macrophage function in CF F508 del backgrounds via antioxidant activity, the inhibition of TG2, and reduction in Beclin-1 crosslinking and rescue of CFTR function [16]. Studies have also shown inhibition of IL-1β and reactive oxygen species (ROS) production, key triggers of inflammation in CF [10–15,29–31]. In this trial, cysteamine had the smallest effect in participants on long term macrolides, drugs with well recognised anti-inflammatory properties [30,32]. Macrolides have also been demonstrated to both inhibit [33,34] or promote autophagy [35], perhaps dependent upon cell type, so the exact nature of the interaction between macrolides and cysteamine is worthy of further investigation.

This exploratory trial had several strengths and limitations. The strengths include the multicentre placebo-controlled design and inclusion criteria reflecting the patient population in whom cysteamine would be used if shown to be efficacious. The use of several putative PROMs and different dosing regimens, were further strengths. The exploratory nature of the trial resulted in limitations that will be addressed in future pivotal trials. When the study was designed there were no data available upon which to base sample size calculations for CFRSD-CRISS use in an RCT testing an intervention during exacerbations. Furthermore, because there were no previous data on the use of cysteamine in pulmonary exacerbations this study’s sample size was based upon observed effects of intravenous aminoglycoside on sputum microbial load in pulmonary exacerbations of CF [25]. The current trial differed because there were no stipulations on the previous use of aminoglycosides whereas the trial of Al-Aloul et al excluded patients if they had received any aminoglycoside therapy in the 3 months prior to randomisation [20]. This difference in exclusion criteria may have contributed to increased variability in baseline sputum microbial load. Recruitment into the current trial was discontinued short of the 120 participants primarily because of difficulties recruiting and the Sponsor’s most recent research demonstrating that the antibiotic-potentiating effects of cysteamine are not limited to aminoglycosides, consequently, future studies will have broader antibiotic use in the inclusion criteria [13]. In addition, during the course of this trial, a more palatable and better tolerated formulation of oral cysteamine was developed and this is the form intended for market and therefore future study. Although the cysteamine 450mg BID regimen improved symptom burden using the CFRSD-CRISS (p = 0.050) the many tests of association conducted in this exploratory study means that we cannot exclude the possibility that this is a type I error. There were significant technical issues in the interpretation of microbiology data. Central laboratories did not quantify total counts. Instead only common species were isolated and enumerated on selective media and the combined colony forming units/mL of these organisms presented as total CFU/ml. This probably explains why Gram-negative organisms were not cultured from the sputum of seven participants despite evidence from local laboratories of chronic infection. Additionally, sputum was processed at two separate locations and the methodologies used deviated between sites. Incorrect assumptions regarding the density of sputum also meant that neither site weighed the samples nor were able to provide reliable CFU/unit weight.

The current study indicates that any future studies should, at the very least, include the cysteamine 450mg BID because of an improvement in symptoms. It is notable that while both 450mg BID and TID doses improved the CRISS domains of feverish and chest tightness, only the 450mg BID dose improved total CRISS score. The two 450mg BID and TID treatment groups were well-balanced with regards to age, sex, BMI, FEV_1_, Fuchs’ score, mucolytic use and antibiotic treatment and although there were some differences in the use of macrolides, inhaled aminoglycosides and ivacaftor or lumacaftor/ivacaftor treatment, adjustment for these had minimal impact on the findings. It may be that the higher 450mg TID dose is not clinically beneficial: although cysteamine and its oxidation product cystamine are antioxidants [13], in the presence of transition metals (typical of sputum in CF) cysteamine is oxidised in a dose-dependent manner releasing potentially damaging free radicals and hydrogen peroxide [36]. Given the small size of the treatment groups and the number of tests of association performed, a possible explanation for the differences between the 450mg BID and TID doses are either type I or type II errors. The lack of signal in 300mg TID compared with 450mg BID is also of interest, and whilst the trial was not designed to compare the two dose regimen directly, it is likely that maximal blood concentration (Cmax) is a very important parameter for cysteamine activity. Cysteamine has a relatively short half-life and binds plasma components, particularly albumin [37], and hepatic first-pass metabolism is estimated to be 40% [38], therefore higher individual doses are more likely to reach a threshold for observable activity. Further studies are required in which the 450 mg regimens will be directly compared.

In conclusion: this multicentre exploratory RCT has provided valuable information that will inform the design of future confirmatory and pivotal trials of cysteamine as an adjunctive treatment in pulmonary exacerbations of CF. Cysteamine appeared to be safe and well-tolerated. Within the limitations of this exploratory study, of the five potential cysteamine dosing regimens tested, the cysteamine 450mg twice and three times daily warrant further investigation in suitably powered trials of pulmonary exacerbations of CF with the PROM CFRSD-CRISS and blood leukocyte count being prioritized as outcome measures.

## Supporting information

S1 Checklist (DOC)

S1 Table Change from baseline to day 21 for selected outcomes. (DOCX)

S2 Table Summary of participant reported adverse events (AEs) by study group. (DOCX)

S1 Appendix (DOCX)

S1 File (PDF)
