# Supplementary material for: Oral cysteamine as an adjunct treatment in cystic fibrosis pulmonary exacerbations: An exploratory randomized clinical trial
Source: PLoS One. 2020 Dec 28;15(12):e0242945. doi: 10.1371/journal.pone.0242945 (PMC7769283; doi:10.1371/journal.pone.0242945)
Supplement: S1 Table — (DOCX) [file pone.0242945.s002.docx]

**S1 table.** **Change from Baseline to day 21 for selected outcomes.**

|  | Placebo  n=17 | Cysteamine dose | | | | |
| --- | --- | --- | --- | --- | --- | --- |
|  |  | 450mg QD  n=11 | 150mg TID  n=15 | 450mg BID  n=15 | 300mg TID  n=16 | 450mg TID  n=15 |
| *Sputum bacteria** |  |  |  |  |  |  |
| Baseline,  mean (SD) | 6.67 (2.09) | 4.76 (3.66) | 6.43 (2.34) | 7.08 (2.50) | 7.21 (2.00) | 5.89 (2.62) |
| Day 21 Change from Baseline | -0.16 (1.28) | -1.22 (2.76) | -0.26 (1.64) | -1.04 (2.89) | -0.87 (1.68) | 0.93 (2.35) |
| Mean (SD) |  | -1.57  (-3.32, 0.18) | 0.04  (-1.55, 1.62) | -0.42  (-2.03, 1.18) | -0.25  (-1.96, 1.46) | 0.97  (-0.59, 2.54) |
| P |  | 0.077 | 0.964 | 0.602 | 0.770 | 0.220 |
| *CFRSD CRISS* |  |  |  |  |  |  |
| Baseline,  mean (SD) | 48.5 (10.6) | 48.9 (12.14) | 47.5 (8.10) | 54.3 (13.13) | 51.0 (10.93) | 56.1 (8.82) |
| Day 21 Change from Baseline  Mean (SD) | -18.3 (11.7) | -23.2 (13.36) | -15.8 (11.68) | -19.9  (19.23) | -12.9  (6.37) | -22.0 (17.71) |
| Day 21 LSMD (cysteamine – placebo)  mean (95% CI) |  | -3.94  (-13.9, 6.00) | 2.55  (-6.65, 11.8) | 0.82  (-8.20, -9.84) | 6.43  (-2.71, 15.6) | -1.64  (-10.7, 7.42) |
| P |  | 0.432 | 0.582 | 0.857 | 0.165 | 0.718 |
| *White blood count x10^9^/l* |  |  |  |  |  |  |
| Baseline,  mean (SD) | 12.42 (4.19) | 10.72 (3.27) | 12.14 (3.93) | 10.69 (2.76) | 10.76 (3.40) | 13.54 (3.32) |
| Day 21 Change from Baseline  Mean (SD) | -1.87 (4.61) | -1.76 (2.67) | -1.88 (3.09) | -1.76 (3.32) | -0.13 (2.59) | -3.32 (3.63) |
| Day 21 LSMD (cysteamine – placebo)  mean (95% CI) |  | -0.42  (-2.85, 2.01) | 0.31  (-1.94, 2.57) | -0.37  (-2.58, 1.85) | 0.88  (-1.44, 3.20) | -0.40  (-2.65, 1.84) |
| P |  | 0.730 | 0.782 | 0.740 | 0.452 | 0.184 |
| *FEV_1_ % predicted* |  |  |  |  |  |  |
| Baseline,  mean (SD) | 41.5 (15.31)) | 39.4 (19.81) | 48.0 (18.26) | 38.2 (21.8) | 36.8 (17.7) | 31.9 (17.2) |
| Day 21 Change from Baseline  Mean (SD) | 9.5 (13.5) | 4.7 (5.03) | 6.46 (7.25) | 8.86 (10.7) | 6.38 (8.18) | 6.29 (11.5) |
| Day 14 LSMD (cysteamine – placebo)  mean (95% CI) |  | -4.80  (-12.9, 3.30) | -2.89  (-10.4, 4.63) | -0.46  (-7.83, 6.91) | -2.99  (-10.5, 4.51) | -3.19  (-10.5, 4.16) |
| P |  | 0.241 | 0.446 | 0.900 | 0.429 | 0.390 |

* Log10-Gram-Negative Sputum Bacterial Load (CFU/mg).

QD = once daily; BID = two times daily; TID = three times daily; TDD = total daily dose;

LSMD = least square mean difference;

Analysis using mixed model for repeated measures (MMRM).
