# Supplementary material for: Oral cysteamine as an adjunct treatment in cystic fibrosis pulmonary exacerbations: An exploratory randomized clinical trial
Source: PLoS One. 2020 Dec 28;15(12):e0242945. doi: 10.1371/journal.pone.0242945 (PMC7769283; doi:10.1371/journal.pone.0242945)
Supplement: S2 Table — (DOCX) [file pone.0242945.s003.docx]

**S2 table. Summary of participant reported adverse events (AEs) by study group.**

|  | Placebo  n=17 | Cysteamine dose | | | | |
| --- | --- | --- | --- | --- | --- | --- |
|  |  | 450mg QD  n=11 | 150mg TID  n=15 | 450mg BID  n=15 | 300mg TID  n=16 | 450mg TID  n=15 |
| Number of AEs | 30 | 29 | 40 | 33 | 45 | 36 |
| Number of SAEs | 1 | 1 | 2 | 1 | 0 | 1 |
| ≥1 AE n(%) | 9 (53%) | 10 (91%) | 11 (73%) | 12 (80%) | 10 (63%) | 13 (87%) |
| AE by severity  Mild  Moderate  Severe | 7 (41%)  2 (12%)  0 | 6 (55%)  4 (36%)  0 | 5 (33%)  6 (40%)  0 | 6 (40%)  5 (33%)  1 (7%) | 4 (25%)  5 (31%)  1 (6%) | 7 (47%)  6 (40%)  0 |
| AE leading to drug discontinuation | 1 (6%) | 1 (9%) | 1 (7%) | 1 (7%) | 1 (6%) | 1 (7%) |
|  |  |  |  |  |  |  |
| Nausea | 0 | 3 (27%) | 5 (33%) | 3 (20%) | 5 (31%) | 3 (20%) |
| Headache | 1 (6%) | 5 (46%) | 1 (7%) | 3 (20%) | 2 (13%) | 1 (7%) |
| Vomiting | 0 | 2 (18%) | 2 (13%) | 2 (13%) | 2 (13%) | 1 (7%) |
| Insomnia | 0 | 0 | 2 (13%) | 0 | 1 (6%) | 2 (13%) |
| Rash | 0 | 0 | 1 (7%) | 1 (6.7) | 3 (18.8) | 0 |
| Arthralgia | 0 | 1 (9%) | 1 (7%) | 1 (6.7) | 0 | 1 (7%) |
| Decreased appetite | 0 | 0 | 1 (7%) | 0 | 2 (12.5) | 1 (7%) |
| Haemoptysis | 2 (12%) | 0 | 1 (7%) | 1 (7%) | 2 (12.5) | 0 |
| Oropharyngeal pain | 0 | 1 (9%) | 1 (7%) | 0 | 1 (6%) | 1 (7%) |
| Abdominal pain | 1 (6%) | 0 | 1 (7%) | 0 | 2 (13%) | 0 |
| Breath odour | 0 | 0 | 0 | 1 (7%) | 0 | 2 (13%) |

AE adverse event, SAE serious adverse event. QD = once daily; BID = two times daily; TID = three times daily; TDD = total daily dose;
