# Supplementary material for: Oral cysteamine as an adjunct treatment in cystic fibrosis pulmonary exacerbations: An exploratory randomized clinical trial
Source: PLoS One. 2020 Dec 28;15(12):e0242945. doi: 10.1371/journal.pone.0242945 (PMC7769283; doi:10.1371/journal.pone.0242945)
Supplement: S1 Appendix — (DOCX) [file pone.0242945.s004.docx]

**Appendix** – **Institutional Review Board (IRB) US and Institutional Ethics Committees (IEC) EU Information for CARE-CF-1 trial**

Protocol title: A Randomized, Double-Blind, Parallel Group, Placebo-Controlled Study Investigating the Optimal Dose Regimen, Efficacy, and Safety of Adding Oral Cysteamine in Adult Patients with Cystic Fibrosis (CF) Being Treated for an Exacerbation of CF-associated Lung Disease

Protocol Number: **NBTCS02**

NIH US National library of medicine: **NCT03000348**

Eudract: **2015-004986-99**

| Principal Investigator | Site(s) | Approved by | Approval date (mm/dd/yy) and IRB number if available |
| --- | --- | --- | --- |
| Cori Daines | Banner University of Arizona Medical Center, Tuscon, AZ 85724. | Michelle Salmon, Board member, Aspire IRB | 10/05/2016 |
| Ryan Dougherty | California Pacific Medical Center, San Fransisco, CA 94115 | Western Institutional Review Board, study number 1169263 | 10/21/2016 IRB: 20162382 |
| Rose Franco | Froedtert and the Medical College of Wisconsin Hospitals and Health Partners, Wauwatosa, Milwaukee, WI 53226  Froedtert Hospital Campus  Adult Translational Research Unit | Mark Kostic, Chair, Institutional Review Board #2, Medical College of Wisconsin and Froedtert Hospital | 02/13/2017 |
| Benjamin Kopp | Nationwide Children’s Hospital, Columbus, OH 43205 | Karen A. White, Chair Institutional Review Board, Nationwide Children’s Hospital | 11/01/2016 |
| Jorge Lascano | University of Florida, Health Shands Medical Plaza, Gainesville, FL 32610  University of Florida, Clinical Research Centre, Gainesville, FL 32610  University of Florida, Health Shands Hospital, FL 32608 | Western Institutional Review Board, study number 1171514 | 01/20/2017 IRB: 20162382 |
| Daniel Layish | Central Florida Pulmonary Group, Altamonte Springs, FL 32701  Central Florida Pulmonary Group, Orlando, FL 32803 | Rebecca Clarke, Board member, Aspire IRB | 06/21/2016 |
| Thomas Smith | Albany Medical College, Albany, NY 12208  Albany Medical Hospital, Albany, NY 12208 | Bonnie Brookshire, Board member, Aspire IRB | 10/31/2016 |

Table 1: **List of US Principal investigators, study sites and IRB approvals**

| Principal Investigator | Site(s) | Approved by | Approval date (DD/MM/YYY) and Number (if available) |
| --- | --- | --- | --- |
| Graham Devereux | Royal Aberdeen Children’s Hospital, Aberdeen, UK AB25 2ZG | Alison Stewart, Research and Development Directorate, NHS Grampian | 05/12/2016 IRAS number: 195249 |
| simon doe | Newcastle Upon Tyne Hospitals NHS Foundation Trust, Newcastle, UK NE3 3HD | Gemma Whitehead,  Research and Development Officer, Newcastle Joint Research Office | 22/02/2017 IRAS number: 195249 |
| alastair innes | Western General Hospital, Edinburgh, UK EH4 2XU | Fiona McArdle, Deputy R&D Director, NHS Lothian | 17/02/2017 |
| gordon mcgregor | NHS Greater Glasgow and Clyde | Ross Nicol, Research Coordinator, NHS Greater Glasgow and Clyde Board | 12/12/2016 |
| lorna murray | Raigmore Hospital, Inverness, UK, IV2 3UJ | Frances Hinds, Research, Development and Innovation Manager, NHS Highland | 09/11/2017 |
| Daniel pekham | Leeds Teaching Hospitals NHS Trust, Leeds, UK LS1 3EX | Donna Johnstone, Research and Innovation Manager, Leeds Teaching Hospitals NHS Trust | 30/10/2017 |
| Vincencida Lucidi | Ospedale Pediatrico Bambino Gesu – Roma, Italy | Chiara Mennini, Segretario, Tecnico Scientifica, Il Comitato Etico, Ospedale Pediatrico Bambino Gesu - Roma | 07/04/2017 |
| sonia volpi | University hospital of Verona | Anna Fratucello, Chair of the Ethics Committee, Ethics committee for clinical trials  of the province of Verona and Rogivo, Integrated University Hospital Authority of Verona | 11/01/2017 |

Table 2**: List of EU Principal investigators, study sites and IEC approvals**
