# Supplementary material for: Oral cysteamine as an adjunct treatment in cystic fibrosis pulmonary exacerbations: An exploratory randomized clinical trial
Source: PLoS One. 2020 Dec 28;15(12):e0242945. doi: 10.1371/journal.pone.0242945 (PMC7769283; doi:10.1371/journal.pone.0242945)
Supplement: S1 File — (PDF) [file pone.0242945.s005.pdf]

CLINICAL RESEARCH PROTOCOL

**DRUG:**	Cysteamine
**STUDY NUMBER(S):**	NBTCS02
**PROTOCOL(S) TITLE:**	A Randomized, Double-Blind, Parallel Group, Placebo-Controlled Study Investigating the Optimal Dose Regimen, Efficacy, and Safety of Adding Oral Cysteamine in Adult Patients with Cystic Fibrosis (CF) Being Treated for an Exacerbation of CF-associated Lung Disease
**IND NUMBER:**	127409
**EudraCT NUMBER:**	2015-004986-99
**SPONSOR:**	NovaBiotics, Ltd.<br>Cruickshank Building<br>Craibstone, Aberdeen AB21 9TR<br>United Kingdom
**ORIGINAL PROTOCOL DATE:**	16 November 2015
**AMENDMENT 1:**	07 March 2016
**AMENDMENT 2:**	21 July 2016
**AMENDMENT 2-UK:**	05 October 2016
**AMENDMENT 2-IT:**	02 November 2016
**AMENDMENT 3:**	18 October 2017

CLINICAL PROTOCOL APPROVAL FORM

Protocol Title: A Randomized, Double-Blind, Parallel Group, Placebo-Controlled Study Investigating the Optimal Dose Regimen, Efficacy, and Safety of Adding Oral Cysteamine in Adult Patients with Cystic Fibrosis (CF) Being Treated for an Exacerbation of CF-associated Lung Disease

Study No: NBTCS02

Original Protocol Date: 16 November 2015

Amendment 1: 07 March 2016

Amendment 2: 21 July 2016

Amendment 2-UK: 05 October 2016

Amendment 2-IT: 02 November 2016

Amendment 3: 30 August 2017

This study protocol was subject to critical review and has been approved by the Sponsor's appropriate protocol review committee. The information contained in this protocol is consistent with:

- The current risk-benefit evaluation of the study drug.
- The moral, ethical and scientific principles governing clinical research as set out in the Declaration of Helsinki, and principles of Good Clinical Practice (GCP) as described in the International Conference on Harmonisation (ICH) guidelines and according to applicable local requirements.

The Investigator will be supplied with details of any significant or new findings, including adverse events relating to treatment with the investigational product.

**Name and Title**	**Signature**	**Date**
Sponsor Representative:<br>Deborah O'Neil, PhD<br>NovaBiotics, Ltd<br>Chief Executive Officer & Chief Scientific Officer		
Coordinating Investigator:<br>Prof. Graham Devereux<br>Aberdeen Royal Infirmary<br>Professor of Respiratory Medicine		
Biostatistics:<br>Elizabeth Ludington, PhD<br>Agility Clinical, Inc.<br>Vice President, Biometrics		

NBTCS02

A Randomized, Double-Blind, Parallel Group, Placebo-Controlled Study Investigating the Optimal Dose Regimen, Efficacy, and Safety of Adding Oral Cysteamine in Adult Patients with Cystic Fibrosis (CF) Being Treated for an Exacerbation of CF-associated Lung Disease

Investigator Statement

I have read the protocol, including all appendices, and I agree that it contains all of the necessary information for me and my staff to conduct this study as described. I will conduct this study as outlined herein, in accordance with the regulations stated in the International Conference on Harmonisation (ICH) guidelines, as well as local regulations, and will make a reasonable effort to complete the study within the time designated.

I will provide all study personnel under my supervision with copies of the protocol and any amendments, and access to all information provided by NovaBiotics, Ltd or specified designees. I will discuss the material with them to ensure that they are fully informed about cysteamine and the study.

______________________________	________________
Principal Investigator Signature	Date
______________________________	________________
Printed Name	Site Number

Study Personnel & Contacts

Sponsor:	NovaBiotics, Ltd<br>Cruickshank Building<br>Craibstone, Aberdeen AB21 9TR<br>United Kingdom<br>Primary Contact: Deborah O'Neil<br>email: deborah@novabiotics.co.uk<br>Telephone: +44 1224 711 377
Coordinating Investigator	Prof. Graham Devereux<br>Professor of Respiratory Medicine<br>Chest Clinic C<br>Aberdeen Royal Infirmary<br>Foresterhill, Aberdeen AB25 2ZN<br>United Kingdom<br>Telephone: +44 1224 438 476
Global Project Management, Project Management & Monitoring for European Union	PSR Group B.V.<br>Crown Business Center Schiphol<br>Planetenweg 5<br>2132 HN Hoofddorp<br>The Netherlands<br>Telephone: +31 23 55 63 220
Data Management	PSR Group B.V.<br>Crown Business Center Schiphol<br>Planetenweg 5<br>2132 HN Hoofddorp<br>The Netherlands<br>Telephone: +31 23 55 63 220
Project Management & Monitoring for United States of America	Agility Clinical, Inc.<br>6005 Hidden Valley Road, Suite 170<br>Carlsbad, CA 92011<br>United States of America<br>Telephone: +1 760 268 8200
Biostatistics	Agility Clinical, Inc.<br>6005 Hidden Valley Road, Suite 170<br>Carlsbad, CA 92011<br>United States of America<br>Telephone: +1 760 268 8200

Medical Monitor & SAE Reporting	Product Life Group.<br>St. John's Innovation Park<br>Cambridge CB4 0WS<br>United Kingdom<br>Telephone: +44 1223 40 2660

Study Synopsis

**Name of Study Compound**	Cysteamine
**Protocol Number**	NBTCS02
**Protocol Title**	A Randomized, Double-Blind, Parallel Group, Placebo-Controlled Study Investigating the Optimal Dose Regimen, Efficacy, and Safety of Adding Oral Cysteamine in Adult Patients with Cystic Fibrosis (CF) Being Treated for an Exacerbation of CF-associated Lung Disease
**Phase of Development**	2
**Primary Study Objectives**	• To determine the optimal dose and regimen of cysteamine in comparison with placebo in patients with exacerbations of CF-associated lung disease<br>• To further evaluate the safety and tolerability of cysteamine in patients with exacerbations of CF-associated lung disease
**Secondary Study Objectives**	• To characterize clinical benefit arising from use of cysteamine in exacerbations of CF-associated lung disease in the Cystic Fibrosis Respiratory Symptom Diary (CFRSD)–Chronic Respiratory Infection Symptom Score (CRISS), Jarad and Sequerios Symptom Score, Cystic Fibrosis Questionnaire-Revised (CFQ-R) questionnaires, and Patient Global Assessment of Exacerbation<br>• Determine the effects of treatment with cysteamine on an exacerbation of CF-associated lung disease for each of the following:<br>o Sputum IL8 and neutrophil elastase levels<br>o Forced expiratory volume in the first second (FEV1)<br>o Weight and body mass index (BMI)<br>o C-reactive protein (CRP)<br>o Blood leukocyte count<br>o Assessment of blood and sputum cysteamine levels

**Study Design**	This is a multicenter, double-blind, randomized, placebo-controlled, 6-arm study to investigate the optimal dose regimen, efficacy, and safety of cysteamine in the treatment of adult patients with CF who are experiencing an exacerbation of CF-associated lung disease.<br>Patients will be screened for the study and eligible patients will be randomized to receive either cysteamine or placebo as add-on therapy to their standard of care treatment for CF-associated lung disease. Approximately 120 patients with CF will be randomized in a 1:1:1:1:1:1 ratio to the following total daily dose groups of cysteamine or placebo: 450 mg as one dose (20 patients): 450 mg as 3 doses of 150 mg (20 patients): 900 mg as 2 doses of 450 mg (20 patients): 900 mg as 3 doses of 300 mg (20 patients): 1,350 mg as 3 doses of 450 mg (20 patients): placebo (20 patients).<br>During the treatment period, patients will take study drug daily for 14 days, in accordance with their treatment assignment, and will be evaluated for the study on Days 7, 14, and 21. Patients who discontinue prior to Day 21 will be requested to complete the procedures for Day 21/End of Study. During the study, patients will be evaluated for the following efficacy parameters: sputum microbial load, sputum IL8 and neutrophil elastase levels, FEV1, weight, CRP, blood leukocyte count, assessment of blood and sputum cysteamine levels, and the following health-related questionnaires/patient-reported outcome measurements (PROMs): Cystic Fibrosis Respiratory Symptom Diary – Chronic Respiratory Infection Symptom Scale (CFRSD-CRISS), Jarad and Sequeiros Symptom Score Questionnaire, Cystic Fibrosis Questionnaire – Revised (CFQ-R), and Patient Global Assessment of Exacerbation.<br>Patient safety will be evaluated by adverse events (AEs), laboratory assessments (chemistry, hematology, and urinalysis), physical examinations, and vital signs. Additionally, a Data Safety Monitoring Board (DSMB) will review the study at periodic intervals, as specified in the DSMB Charter.

Study Schematic

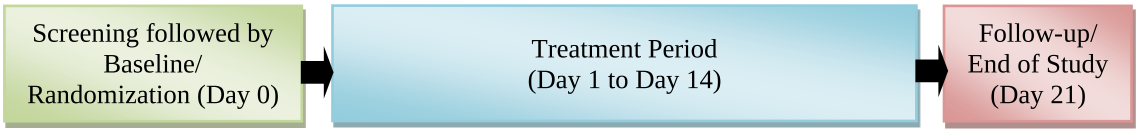

**Number of Patients**	Approximately 120 patients will be randomized in the study.
**Study Population**	Adult patients with CF-associated lung disease infected with a Gram-negative organism(s) (e.g., *Pseudomonas, Burkholderia, Stenotrophomonas*) who experience an exacerbation of their lung disease.

<table>
<tr><td>Number of Study Sites</td><td>Up to 20 study centers located in Europe and up to 20 study centers located in the United States of America (USA) will participate in this study.</td></tr>
<tr><td>Treatments</td><td>Cysteamine (as mercaptamine bitartrate) will be provided as 150 mg hard capsules for oral administration. Placebo will be provided as matching hard capsules.
Patients will be randomly assigned to one of the following treatment groups:
<table>
<tr><th rowspan="2">Treatment Group</th><th rowspan="2">Total Daily Dose</th><th colspan="3">Dosing Regimen</th></tr>
<tr><th>Morning</th><th>Mid-Day</th><th>Evening</th></tr>
<tr><td>A</td><td>cysteamine 450 mg</td><td>3 cysteamine 150 mg capsules</td><td>3 placebo capsules</td><td>3 placebo capsules</td></tr>
<tr><td>B</td><td>cysteamine 900 mg</td><td>3 cysteamine 150 mg capsules</td><td>3 placebo capsules</td><td>3 cysteamine 150 mg capsules</td></tr>
<tr><td>C</td><td>cysteamine 1,350 mg</td><td>3 cysteamine 150 mg capsules</td><td>3 cysteamine 150 mg capsules</td><td>3 cysteamine 150 mg capsules</td></tr>
<tr><td>D</td><td>Placebo</td><td>3 placebo capsules</td><td>3 placebo capsules</td><td>3 placebo capsules</td></tr>
<tr><td>E</td><td>cysteamine
450 mg</td><td>1 cysteamine
150 mg capsule
2 placebo capsules</td><td>1 cysteamine
150 mg capsule
2 placebo capsules</td><td>1 cysteamine
150 mg capsule
2 placebo capsules</td></tr>
<tr><td>F</td><td>cysteamine
900 mg</td><td>2 cysteamine
150 mg capsules
1 placebo capsule</td><td>2 cysteamine 150 mg capsules
1 placebo capsule</td><td>2 cysteamine 150 mg capsules
1 placebo capsule</td></tr>
</table></td></tr>
<tr><td>Duration of Participation</td><td>Following randomization, each eligible patient will participate in the study for 3 weeks, including 2 weeks of treatment and 1 week of follow-up.</td></tr>
<tr><td>Inclusion Criteria</td><td>Patients must meet all of the following criteria to be considered eligible to participate in the study:
1) CF-associated lung disease with documented history of chronic infection with Gram-negative organism(s)
2) Established patient of the Principal Investigator’s CF Multi-Disciplinary Team (MDT)
3) Age ≥18 years
4) Weight >40 kg
5) FEV1 >30% of predicted within the 6 months prior to study exacerbation</td></tr>
</table>

	6) At the baseline visit: experiencing a new exacerbation of CF-associated lung disease (based on Investigator assessment of ≥4 symptoms present on the Fuchs' criteria) requiring treatment that includes an aminoglycoside antibiotic (see Section 6.4)<br>7) Females of childbearing potential will be included if they are either sexually inactive (sexually abstinent for 14 days prior to the first study drug dose continuing through 28 days after the last study drug dose, or using one of the following highly effective contraceptive (i.e. results in <1% failure rate when used consistently and correctly) methods in this trial:<br>a. intrauterine device (IUD);<br>b. surgical sterilization of the partner (vasectomy for 6 months minimum);<br>c. combined (estrogen or progestogen containing) hormonal contraception associated with the inhibition of ovulation (either oral, intravaginal, or transdermal);<br>d. progestogen only hormonal contraception associated with the inhibition of ovulation (either oral, injectable, or implantable);<br>e. intrauterine hormone releasing system (IUS);<br>f. bilateral tubal occlusion.<br>8) Females of childbearing potential agree to remain sexually inactive or to keep the same birth control method for at least 28 days following the last dose.<br>9) A female of non-childbearing potential must have undergone one of the following sterilization procedures at least 6 months prior to the first study drug dose:<br>a. hysteroscopic sterilization;<br>b. bilateral tubal ligation or bilateral salpingectomy;<br>c. hysterectomy;<br>d. bilateral oophorectomy;<br>or be postmenopausal with amenorrhea for at least 1 year prior to the first study drug dose and follicle stimulating hormone (FSH) serum levels consistent with postmenopausal status.<br>10) A non-vasectomized male subject agrees to use a condom with spermicide or abstain from sexual intercourse during the study until 90 days beyond the last dose of study medication and the female partner agrees to comply with inclusion 7 or 9. For a vasectomized male who has had his vasectomy 6 months or more prior to study start, it is required that they use a condom during sexual intercourse. A male who has been vasectomized less than 6 months prior to study start must follow the same restrictions as a non-vasectomized male.<br>11) If male, agrees not to donate sperm from the first study drug dose until 90 days after dosing.

	12) Willing and able to comply with all protocol requirements and procedures, including induction of sputum, if necessary<br><br>13) Willing and able to provide signed and dated informed consent
**Exclusion Criteria**	Patients who meet any of the following criteria will be excluded from participation in the study:<br>1) Hypersensitive to cysteamine or to any of the excipients<br>2) Hypersensitive to penicillamine<br>3) Transplant recipient<br>4) Participation in any other interventional clinical research study (participation in observational studies is not exclusionary) within 30 days of Baseline (Day 0), and any planned participation in an interventional clinical research study for the duration of this study<br>5) If female, pregnancy, planned pregnancy, or breast-feeding<br>6) Any other significant disease/disorder which, in the Investigator's opinion, either puts the patient at risk due to study participation, or may influence the results of the study or the patient's ability to participate in the study
**Efficacy Assessments**	Efficacy parameters collected include the following: sputum microbial load, sputum IL8 and neutrophil elastase levels, FEV1, weight, CRP, blood leukocyte count, assessment of blood and sputum cysteamine levels, and the following health-related questionnaires/PROMs: CFRSD–CRISS, Jarad and Sequeiros Symptom Score Questionnaire, CFQ-R, and Patient Global Assessment of Exacerbation.
**Safety Assessments**	Safety parameters collected include: AEs, serious adverse events (SAEs), vital signs, physical examinations, and laboratory parameters (chemistry, hematology, and urinalysis).
**Sample Size Justification**	A total of approximately 120 patients with CF being treated for an exacerbation of CF-associated lung disease will be enrolled in the study: 20 patients in each group. The sample size of 120 patients was selected empirically without a formal statistical assumption. The sample size selection is considered to be appropriate to determine the optimal dose and regimen based on evidence of efficacy and acceptable safety and tolerability profile as well as establish point estimates and variability for efficacy endpoints for future evaluation.<br><br>A sample size of 20 patients in each group will have approximately 80% power to detect a 1.2 log reduction over placebo, assuming a standard deviation of 1.31 in the reduction of sputum bacterial load, based on a two-sided, two-sample t-test at the 5% level of significance. This estimated standard deviation is that reported for a 2-week study of CF patients with *Pseudomonas aeruginosa* who were treated during exacerbations with 2 weeks of intravenous tobramycin (Al-Aloul et al, 2014).

**Statistical Methods and Types of Analyses**	The primary objective of this study, to determine the optimal dose and regimen of cysteamine in patients with exacerbations of CF-associated lung disease, will be investigated through a combination of safety, efficacy, and microbiology endpoints. Though statistical comparisons between treatment groups with respect to efficacy and microbiology will be performed to help inform the selection of the final dose for further development, safety and tolerability will also be paramount in the selection of the optimal dose.<br>The primary efficacy endpoint for the study is:<br>• Change from baseline (Day 0) in log-transformed sputum bacterial load of gram negative CFU per ml and per mg following a CF exacerbation<br>The secondary efficacy endpoints for the study are:<br>• Change from baseline (Day 0) in patient health-related questionnaires/PROMs (CFRSD-CRISS, Jarad and Sequeiros Symptom Score Questionnaire, and CFQ-R)<br>• Patient Global Assessment of Exacerbation questionnaire<br>• Change from baseline (Day 0) in sputum IL8 and neutrophil elastase levels following a CF exacerbation<br>• Change from baseline (Day 0) in the following:<br>o FEV1<br>o Weight and BMI<br>o CRP<br>o Blood leukocyte count<br>• Assessment of blood and sputum cysteamine levels at Day 14<br><br>The efficacy comparisons at Day 14 of interest will be conducted using a linear mixed model for repeated measures, analysis of variance (ANOVA), and chi-square tests as appropriate. Change from baseline (Day 0) to Day 7 and Day 21 will also be assessed where applicable. All analyses will be conducted using two-sided tests at the alpha=0.05 level of significance. There will be no adjustments for multiple comparisons in the efficacy analyses for this study.<br><br>The data for change from baseline (Day 0) measures will be summarized with descriptive statistics and confidence intervals and will be analyzed via contrast statements from the mixed model for all pairwise and pooled total daily dose (TDD) group comparisons, each at the alpha=0.05 level of significance. Analyses of subgroups and other exploratory linear mixed models may be explored.<br>Continuous variables will be summarized descriptively by the number of patients with non-missing data, mean and standard deviation, median, minimum, and maximum values. Categorical variables will

	be summarized descriptively by their counts and associated percentages.<br>Safety analyses will involve the examination of the descriptive statistics and individual patient listings for any effects of study treatment on clinical safety. Summaries will be prepared by treatment group and TDD group.<br>Treatment-emergent adverse event (TEAE) summaries will include the overall incidence (by system organ class and preferred term), events by maximum intensity, events by relationship to study treatment, events leading to discontinuation of study drug, and SAEs.<br>Vital signs and laboratory parameters (hematology, chemistry, and urinalysis) will be summarized descriptively by treatment. Actual and change from baseline (Day 0) data will be calculated and summarized.<br>Each potentially efficacious dose will also be assessed from a safety and tolerability perspective based on assessment of AEs, laboratory values, and vital signs. Potential doses for future development will consist of one or more doses that are both potentially efficacious and have demonstrated an adequate safety profile.

TABLE OF CONTENTS

CONTENTS

Study Personnel & Contacts 4
1 INTRODUCTION AND RATIONALE 18
1.1 Background 18
1.1.1 Cystic Fibrosis (CF) 18
1.1.2 Cysteamine 19
1.1.3 Rationale for Using Cysteamine in CF 19
1.1.4 Clinical Experience with Cysteamine in Patients with CF 20
2 STUDY OBJECTIVES 21
2.1 Main Objectives 21
2.2 Additional Objectives .... Error! Bookmark not defined.
3 STUDY ENDPOINTS 22
4 STUDY PLAN 22
4.1 Study Design 22
4.2 Study Schematic 23
4.3 Schedule of Assessments 24
5 RANDOMIZATION AND BLINDING 25
5.1 Randomization and Blinding 25
5.2 Emergency Unblinding 25
6 POPULATION 26
6.1 Number of Patients and Study Centers 26
6.2 Inclusion Criteria 26
6.3 Exclusion Criteria 28
6.4 Fuchs' Criteria 28
6.5 Deviation from Inclusion/Exclusion Criteria 29
7 STUDY CONDUCT 29
7.1 General Instructions 29
7.2 Study Procedures by Time Point 29
7.2.1 Screening 29
7.2.2 Treatment Period 30
7.2.3 Follow-Up 32
7.2.4 Unscheduled Visits 32
7.3 Premature Discontinuation 32
8 STUDY DRUG 33
8.1 Cysteamine & Placebo Descriptions 33
8.2 Packaging, Shipment, and Storage 34
8.3 Dose and Administration 34
8.4 Accountability and Compliance 34
9 DESCRIPTION OF STUDY PROCEDURES 35
9.1 Sputum Analysis 35
9.2 FEV1 35
9.3 Health-related Questionnaires/Patient-Reported Outcome Measures (PROMs) 36

9.3.1 Cystic Fibrosis Respiratory Symptom Diary (CFRSD)–Chronic Respiratory Infection Symptom Scale (CRISS) 36
9.3.2 Jarad and Sequeiros Symptom Score Questionnaire 36
9.3.3 Cystic Fibrosis Questionnaire – Revised (CFQ-R) 36
9.3.4 Patient Global Assessment of Exacerbation 36
9.4 Physical Examinations 36
9.5 Vital Signs, Height, and Weight 37
9.6 Laboratory Testing 37
9.6.1 Clinical Laboratory Tests 38
9.6.2 Cysteamine plasma and sputum level sampling 38
10 Concomitant Medications, Non-Medication Therapies, and Study Restrictions 39
10.1 Prohibited Foods and Medications 39
10.1.1 Restricted Foods 39
10.1.2 Restricted Medications 39
11 ADVERSE EVENTS 39
11.1 Definition of Adverse Events 39
11.2 Documenting Adverse Events 40
11.3 Assessment of Severity 40
11.4 Assessment of Causality 41
11.5 Adverse Event Outcome 41
11.5.1 Withdrawal Due to Adverse Event 42
12 SERIOUS ADVERSE EVENT 42
12.1 Definition of Serious Adverse Event 42
12.2 SAE Expectedness 43
12.3 Reporting Serious Adverse Events 44
12.3.1 Overdose 44
12.3.2 Pregnancy 44
13 STATISTICS 45
13.1 Analysis Populations 45
13.2 General Procedures 45
13.3 Sample Size 45
13.4 Statistical Methods 46
13.4.1 Demographic and Baseline Characteristics 46
13.4.2 Efficacy Analyses 46
13.4.3 Safety Analyses 47
13.5 Interim Analysis 47
14 ETHICS AND RESPONSIBILITIES 47
14.1 Good Clinical Practice 47
14.2 Data Safety Monitoring Board (DSMB) 48
14.3 Institutional Review Board (IRB)/Ethics Committee (EC) 48
14.4 Informed Consent 48
14.5 Records Management and Study Monitoring 49
14.6 Source Documentation and Data Collection 49
14.7 Study Files and Record Retention 50
14.8 Declaration of End of Clinical Study 50
14.9 Registration of Clinical Study 50
15 AUDITING 50
16 AMENDMENTS 50

17 FINANCING AND INSURANCE..51
18 STUDY REPORT AND PUBLICATIONS..51
19 STUDY DISCONTINUATION 51
20 CONFIDENTIALITY 51
21 REFERENCES 52
22 APPENDICES...54
22.1 APPENDIX A – Cystic Fibrosis Respiratory Symptom Diary (CFRSD) – Chronic Respiratory Infection Symptom Scale (CRISS)...54
22.2 APPENDIX B – Jarad and Sequeiros Symptom Score Questionnaire 60
22.3 APPENDIX C – Cystic Fibrosis Questionnaire – Revised (CFQ-R) 61
22.4 APPENDIX D – Patient Global Assessment of Exacerbation 64
22.5 APPENDIX E – Declaration of Helsinki...65

LIST OF ABBREVIATIONS

AE	Adverse Event
AR	Adverse Reaction
ATS	American Thoracic Society
BMI	Body Mass Index
°C	Celsius
CF	Cystic Fibrosis
CFQ-R	Cystic Fibrosis Questionnaire – Revised
CFR	Code of Federal Regulations
CFRSD	Cystic Fibrosis Respiratory Symptom Diary
CFTR	Cystic Fibrosis Transmembrane Regulator
CFU	Colony-Forming Unit
CI	Confidence Interval
CL/F	Oral Clearance
Cmax	Maximum Serum Concentration
eCRF	Electronic Case Report Form
CRISS	Chronic Respiratory Infection Symptom Scale
CRP	C-reactive Protein
DSMB	Data Safety Monitoring Board
EC	Ethics Committee
EDC	Electronic Data Capture
ERS	European Respiratory Society
EU	European Union
eudraCT	European Union Drug Regulating Authorities Clinical Trials
°F	Fahrenheit
FDA	Food and Drug Administration
FEV1	Forced Expiratory Volume in the First Second
GCP	Good Clinical Practice
ICH	International Conference on Harmonisation
IL8	Interleukin 8
IRB	Institutional Review Board
IQR	Interquartile Range
ITT	Intent-to-Treat
IUD	Intrauterine Device
kg	Kilogram(s)
L	Liter(s)
MBC	Minimum Bacterial Concentration
MDT	Multi-Disciplinary Team

mg	Milligram(s)
MIC	Minimum Inhibitory Concentration
MMRM	Mixed Model for Repeated Measures
PP	Per Protocol
PROM	Patient-Reported Outcome Measure
SAE	Serious Adverse Event
SAR	Serious Adverse Reaction
SAP	Statistical Analysis Plan
SD	Standard Deviation
SOP	Standard Operating Procedures
TEAE	Treatment-Emergent Adverse Event
TDD	Total Daily Dose
$T_{1/2}$	Half-Life
Tmax	Time to Maximum Concentration
USA	United States of America
UK	United Kingdom
Vd/F	Volume of Distribution

1 INTRODUCTION AND RATIONALE

1.1 Background

1.1.1 Cystic Fibrosis (CF)

Cystic Fibrosis (CF) is the most common fatal inherited disease in Caucasian populations of European origin. In the United Kingdom (UK), the carrier rate for this autosomal recessive condition is 1 in 25 and disease prevalence is approximately 1 in 2,500. CF affects over 9,000 people in the UK and about 70,000 people globally (UK CF Registry, 2014). CF is associated with reduced life expectancy, with the current median age of death at 26 to 29 years in the UK. CF results from >1,000 mutations in the CF transmembrane regulator (CFTR) gene that codes for a chloride ion channel located in the apical membrane of epithelial cells; the CFTR has also been identified in non-epithelial cells. Although CF affects the pancreas, hepatobiliary tree, and the intestinal and reproductive tracts, by far the most important aspect of the disease is respiratory, with 90% of the morbidity and mortality associated with CF resulting from chronic suppurative lung disease and ultimately respiratory failure (Goss and Quittner, 2007). CFTR mutations result in airway surface liquid dehydration, consequent impairment of mucociliary clearance, bacterial colonization of the airways, and chronic suppurative lung disease. The chronic infection and inflammation of CF lung disease results in daily symptoms such as dyspnea, cough, and sputum production. A characteristic feature of CF is intermittent episodes of acute worsening of symptoms, commonly known as exacerbations. Exacerbations are characterized by increased cough, increased sputum production, increased dyspnea, loss of appetite, weight loss, and lung function decline (Goss and Burns, 2007). Such exacerbations have an adverse impact on patients' quality of life (Hegarty et al, 2009), incur significant healthcare costs (Britto et al, 2002), and are associated with a more rapid loss of lung function (Sanders et al, 2010; Sanders et al, 2011).

Pseudomonas aeruginosa is the most common pathogen in CF (UK CF Registry, 2014), and infection with this organism is characterized by persistence of the bacteria, repeated exacerbations, and an accelerated rate of decline in lung function. Other Gram-negative bacteria can also infect or colonize the lung, probably the most clinically significant of which is the *Burkholderia cepacia* complex. The Gram-negative bacteria that infect patients with CF are intrinsically resistant to many antibiotics, and the prevalence of bacteria with newly acquired resistance has increased with improved life expectancy. Resistance rates in *P. aeruginosa* in the UK have increased dramatically with approximately 40% of patients resistant to two or more antibiotics in one study (Pitt et al, 2003). The development of antibiotic resistance in CF is most likely due to the intensive selective pressure provided by the large amounts of antibiotics used in these patients and the high frequency of hypermutatable *P. aeruginosa* found in CF lung infection (Oliver et al, 2000; Giwercman et al, 1990). In CF, *P. aeruginosa* and the *B. cepacia* complex grow in biofilms and, as such, are much more resistant to antibiotics compared with planktonic-growing cells of the same isolate: minimum inhibitory concentration (MIC) and minimum bacterial concentration (MBC) can be 100- to 1,000- fold greater in biofilms (Stewart and Costerton, 2007). The issue of bacterial antibiotic resistance in CF is compounded by the relatively high incidence of adverse reactions (ARs) to antibiotics that has been reported in 9.5% of CF children and up to 25% in CF adults

(Wills et al, 1998; Pleasants and Samuelson, 1994). The incidence of ARs increases with the number of courses of antibiotics administered (Koch et al, 1991).

The aggressive use of antibiotics to suppress chronic infection and to treat acute exacerbations is one of the mainstays of treatment in CF that has contributed to the increased survival of CF patients. However, the problems of multiple drug resistance and ARs are major clinical issues. This has led to calls for research into new antibiotics and new antibiotic strategies to target the biofilm and to increase the effectiveness of currently available antibiotics (Bals et al, 2011; Hoiby, 2002). Preclinical work indicates that cysteamine, a drug already licensed for another indication, has potentially beneficial actions on biofilms and micro-organisms, and is the basis for the current study.

1.1.2 Cysteamine

Cysteamine is an amino thiol (HSCH2CH2NH2) that is found endogenously in very low plasma levels as a consequence of coenzyme A metabolism (Besouw et al, 2013). Since 1976, cysteamine has been used to treat the lysosomal storage disorder, cystinosis. An immediate release formulation (Cystagon) was licensed for the treatment of cystinosis in the United States of America (USA) in 1994 and in the European Union (EU) in 1997. Adequate treatment of cystinosis with cysteamine reduces the rate of progression to end-stage renal failure, reduces extra renal manifestations, and improves growth.

1.1.3 Rationale for Using Cysteamine in CF

The mucolytic and antimicrobial properties of cysteamine were identified by the study Sponsor (NovaBiotics Ltd). Data obtained in the laboratory and from a clinical pilot study, in which the impact of cysteamine on the *in vitro* rheology and microbial burden of sputum from CF patients was assessed, highlights the therapeutic potential in CF. Cysteamine is a potent mucolytic that is able to break down the sputum produced within the airways of CF patients (Charrier et al, 2014; Devereux et al, 2015). This has the potential to aid sputum expectoration, improve lung function, and reduce the microbial burden in the airways. This mucolytic action may also provide better access for antimicrobial agents to the bacteria within the airways of CF patients. Cysteamine also possesses three key antimicrobial (non-antibiotic) attributes that potentiate the effects of existing antibiotic regimens for CF patients (Charrier et al, 2014; Devereux et al, 2015):

1. Cysteamine kills bacteria commonly associated with CF-associated lung disease.

2. Cysteamine synergizes with other antibiotic agents, broadens their spectrum of activity, delivers a post-antimicrobial effect, and reverses antibiotic resistance (even in multi-drug resistant strains).

3. Cysteamine disrupts the biofilms that bacteria colonize in the airways of CF.

1.1.4 Clinical Experience with Cysteamine in Patients with CF

In a Phase 2 study, oral cysteamine was administered to patients with CF-associated lung disease when not exacerbating to determine whether oral cysteamine is absorbed in adult CF patients and enters the bronchial secretions. Tolerability and clinical outcomes were explored. Ten patients with stable CF associated lung disease were enrolled: median (interquartile ratio [IQR]) age 21.5 (19.8-31.8) years; median (IQR) forced expiratory volume in the first second (FEV1) percent predicted 32% (26-49) predicted; 80% of patients were F508del/F508del and 80% of patients were infected with a Gram-negative organism. Oral cysteamine was commenced at 450 mg once daily, then increased weekly to 450 mg four times daily. The maximal dose was taken for 2 weeks. Serial plasma cysteamine levels were measured for 24 hours after the first dose. Seven of the 10 participants reported ARs typical of cysteamine and two patients discontinued treatment intervention. Following the first 450 mg dose, the mean (SD) maximum serum concentration (Cmax) was 2.86 (1.96) mg/L, the time to maximum concentration (Tmax) was 1.2 (0.7) hours, the half-life ($T_{½}$) was 3.7 (1.7), oral clearance (CL/F) was 89.9 (30.5) L/hour and volume of distribution (Vd/F) was 427 L (129). Cysteamine appeared to accumulate in sputum with a median (IQR) sputum:plasma cysteamine ratio of 4.2 (0.98-8.84). Cysteamine treatment reduced sputum microbial load (0.9 log reduction [95% CI -0.05 to 1.9, p=0.060]) and viscosity with the maximal reduction observed with cysteamine 450 mg three times daily. There was evidence of reduced adherence with the four times daily dosing regimen. Cysteamine therapy was associated with clinically significant reductions in the Cystic Fibrosis Questionnaire-Revised (CFQ-R) quality of life domains of ‘Vitality’ (16 points, p=0.012), ‘Physical Functioning’ (10 points, p=0.058), ‘Eating Disturbance’ (11 points) and ‘Weight’ (10 points).

This study confirmed that oral cysteamine is absorbed and enters the bronchial secretions in patients with CF. The effects of cysteamine on sputum microbial load and sputum viscosity were in line with the preclinical ex vivo data (Charrier et al, 2014; Devereux et al, 2015). The adverse effects on quality of life are potentially clinically important and reinforce the notion that oral cysteamine is likely more suited for use as an adjunct to treatment of exacerbations of CF-associated lung disease where the duration of treatment is relatively short, typically 2 weeks.

The current study builds on the preclinical and clinical data to date by investigating the potential for cysteamine to be used as an adjunct to treat acute exacerbations of CF-associated lung disease, specifically to identify the optimal dose of cysteamine based on effect on sputum microbial load. Oral formulations of cysteamine are available for licensed use in cystinosis, and there are more than 20 years of clinical experience with cysteamine for this indication.

Any future phase 3 trial of cysteamine in acute exacerbations of CF-associated lung disease will require a health-related questionnaire/PROM, and such an outcome measure is currently not available for acute exacerbations in CF. The following four potential health-related questionnaires/PROMs have been identified:

a) The Cystic Fibrosis Respiratory Symptom Diary (CFRSD)–Chronic Respiratory Infection Symptom Score (CRISS) is a patient-completed questionnaire designed to evaluate the effect of treatment on the severity of

symptoms of respiratory infection in patients with CF and a chronic respiratory infection.

b) The Jarad and Sequeiros Symptom Questionnaire (Jarad and Sequieros 2011) is a simple patient-completed questionnaire that assesses and evaluates change in patient symptoms related to different aspects of respiratory function during a CF exacerbation.

c) The CFQ-R is a patient-completed questionnaire to assess the impact of CF and treatment on everyday life, however this may not be ideally suited to the dynamic nature of an acute exacerbation because the CFQ-R enquires about symptoms over previous 2 weeks.

d) The Patient Global Assessment of Exacerbation is a patient-completed questionnaire that assesses the current CF exacerbation in comparison to the previous exacerbation.

Another major aim of this study is to identify health-related questionnaires/PROMs most suited for use in any future study of cysteamine in acute exacerbations of CF-associated lung disease.

2 STUDY OBJECTIVES

2.1 Primary Study Objectives

- To determine the optimal dose and regimen of cysteamine in comparison with placebo in patients with exacerbations of CF-associated lung disease
- To further evaluate the safety and tolerability of cysteamine in patients with exacerbations of CF-associated lung disease

2.2 Secondary Study Objectives

- To characterize clinical benefit arising from use of cysteamine in exacerbations of CF-associated lung disease in the Cystic Fibrosis Respiratory Symptom Diary (CFRSD)- Chronic Respiratory Infection Symptom Score (CRISS), Jarad and Sequerios Symptom Score, Cystic Fibrosis Questionnaire-Revised (CFQ-R) questionnaires, and Patient Global Assessment of Exacerbation
- Determine the effects of treatment with cysteamine on exacerbation of CF-associated lung disease for each of the following:
    - Sputum IL8 and neutrophil elastase leves
    - Forced expiratory volume in the first second (FEV1)
    - Weight and body mass index (BMI)
    - C-reactive protein (CRP)
    - Blood leukocyte count

- Assessment of blood and sputum cysteamine levels

3 STUDY ENDPOINTS

The primary efficacy endpoint for the study is:

- Change from baseline (Day 0) in sputum bacterial load of Gram negative CFU per ml and per mg following a CF exacerbation.

The secondary efficacy endpoints for the study are:

- Change from baseline (Day 0) in patient health-related questionnaires/PROMs (CFRSD CRISS, Jarad and Sequerios Symptom Score Questionnaire, and CFQ-R)
- Patient Global Assessment of Exacerbation questionnaire
- Change from baseline (Day 0) in sputum IL8 and neutrophil elastase levels following a CF exacerbation
- Change from baseline (Day 0) in the following:
  - FEV1
  - Weight and BMI
  - CRP
  - Blood leukocyte count
- Assessment of blood and sputum cysteamine levels at Day 14

4 STUDY PLAN

4.1 Study Design

This is a multicenter, double-blind, randomized, placebo-controlled, 6-arm study to investigate the optimal dose regimen, efficacy, and safety of cysteamine in the treatment of adult patients with CF who are experiencing an exacerbation of CF-associated lung disease.

Patients will be screened for the study and eligible patients will be randomized to receive either cysteamine or placebo as add-on therapy to their standard of care treatment for CF-associated lung disease. Approximately 120 patients with CF will be randomized in a 1:1:1:1:1:1 ratio to the following total daily dose groups of cysteamine or placebo: 450 mg as one dose (20 patients): 450 mg as 3 doses of 150 mg (20 patients): 900 mg as 2 doses of 450 mg (20 patients): 900 mg as 3 doses of 300 mg (20 patients): 1,350 mg as 3 doses of 450 mg (20 patients): placebo (20 patients).

During the treatment period, patients will take study drug daily for 14 days, in accordance with their treatment assignment, and will be evaluated for the study on

Days 7, 14, and 21. Patients who discontinue prior to Day 21 will be requested to complete the procedures for Day 21/End of Study.

During the study, patients will be evaluated for the following efficacy parameters: sputum microbial load, IL8 and neutrophil elastase levels, FEV1, weight, CRP, blood leukocyte count, assessment of blood and sputum cysteamine levels, and the following health-related questionnaires/PROMs: CFRSD-CRISS, Jarad and Sequeiros Symptom Score Questionnaire, CFQ-R, and Patient Global Assessment of Exacerbation.

Patient safety will be evaluated by AEs, laboratory assessments (chemistry, hematology, and urinalysis), physical examinations, and vital signs. Additionally, a Data Safety Monitoring Board (DSMB) will review the study at periodic intervals, as specified in the DSMB Charter.

4.2 Study Schematic

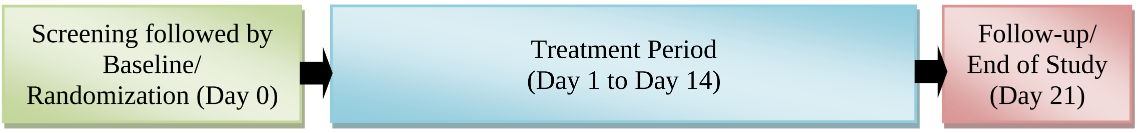

4.3 Schedule of Assessments

Assessment	Screening[a]	Baseline [a] (Day 0)	Day 7 (±1 day)	Day 14 (±1 day)	Day 21 (±1 day) Follow-Up/ End of Study
Written informed consent	X				
Demographic data	X				
Relevant medical/surgical history	X				
Physical examination		X			X
Evaluate Fuchs' criteria		X			
Assessment of eligibility criteria	X	X			
Randomization		X			
Height	X				
Weight	X	X	X	X	X
Vital signs	X	X	X	X	X
FEV1		X	X	X	X
CFRSD–CRISS		X	X	X	X
Jarad and Sequeiros Symptom Score Questionnaire		X	X	X	X
CFQ-R		X	X	X	X
Patient Global Assessment of Exacerbation				X	
Sputum sample[b]		X	X	X	X
Laboratory samples		X	X	X	X
Cysteamine plasma and sputum[b] level sampling				X	
Pregnancy test[c]		X			X
Dispense study drug[d]		X			
Study drug accountability			X	X	
Concomitant medications	X--X				
Non-medication therapies	X--X				
Adverse events[e]		X--------------------------------X			

[a] Screening may occur prior to the start of a CF-associated lung disease exacerbation. Screening and Baseline (Day 0) may be performed as separate visits or as a combined visit. If performed as a combined visit, procedures noted at both visits will only be performed once (i.e., assessment of eligibility criteria, weight, and vital signs). If performed as separate visits, the procedures noted at both visits will be performed at each required visit. Upon determination of eligibility by the Investigator, the patient may be randomized.

[b] Sites may collect separate samples for each required sputum assessment or may collect one sputum sample and split it into either 2 or 3 parts (depending on sample requirements for the visit). The sample will either be split into 2 parts for Day 0, Day 7 and Day 21, and 3 parts for Day 14 or 2 samples will be collected for Day 0, Day 7 and Day 21, or 3 samples will be collected for Day 14. Each part or sample will be placed into a separate container for shipping and analyses, details are included in the Laboratory Manual..

[c] A urine pregnancy test will be performed on all females of childbearing potential. If the urine pregnancy test is positive, a serum pregnancy test will be performed.

[d] Study drug will be dispensed to the patient for self-administration if the patient will be seen on an outpatient basis or at another preferred location (at visits Day 7 and Day 14 only). If the patient will be seen on an inpatient basis, study drug will be administered to the patient daily by qualified site personnel.

[e] AE recording begins at the first dose. Events occurring prior to the first dose will be recorded as medical history.

5 RANDOMIZATION AND BLINDING

5.1 Randomization and Blinding

Cysteamine (as mercaptamine bitartrate) will be provided as 150 mg hard capsules for oral administration.

Patients will be randomly assigned to one of the following treatment groups noted in Table 1.

Table 1: Treatment Groups

Treatment Group	Total Daily Dose	Dosing Regimen		
		**Morning**	**Mid-Day**	**Evening**
A	cysteamine 450 mg	3 cysteamine 150 mg capsules	3 placebo capsules	3 placebo capsules
B	cysteamine 900 mg	3 cysteamine 150 mg capsules	3 placebo capsules	3 cysteamine 150 mg capsules
C	cysteamine 1,350 mg	3 cysteamine 150 mg capsules	3 cysteamine 150 mg capsules	3 cysteamine 150 mg capsules
D	Placebo	3 placebo capsules	3 placebo capsules	3 placebo capsules
E	cysteamine 450 mg	1 cysteamine 150 mg capsule 2 placebo capsules	1 cysteamine 150 mg capsule 2 placebo capsules	1 cysteamine 150 mg capsule 2 placebo capsules
F	cysteamine 900 mg	2 cysteamine 150 mg capsules 1 placebo capsule	2 cysteamine 150 mg capsules 1 placebo capsule	2 cysteamine 150 mg capsules 1 placebo capsule

In order to maintain the study blind, placebo capsules will be taken so that each patient takes 3 capsules 3 times per day (i.e., morning, mid-day, and evening doses).

The randomization schedule will be generated using a computer program and verified for accuracy using strict quality control procedures. Randomization will be centralized.

The investigational site will maintain an inventory of study drug supplied by the Sponsor. Eligible patients will receive a blinded treatment assignment with a unique randomization code based on the randomization list. The assigned randomization code will be captured in the electronic Case Report Form (eCRF).

5.2 Emergency Unblinding

The Study Handbook details the procedures for emergency unblinding. In the event a patient’s treatment assignment is unblinded, this treatment assignment will be kept confidential and shared only as required for patient safety and/or regulatory reporting.

Unblinding should only be performed if knowledge of the treatment assignment is considered relevant for medical care.

6 POPULATION

The patient population will consist of adult patients with CF-associated lung disease infected with a Gram-negative organism(s) (e.g., *Pseudomonas, Burkholderia, Stenotrophomonas*) who experience an exacerbation of their lung disease.

6.1 Number of Patients and Study Centers

Approximately 120 patients will be randomized in the study.

Patients will be enrolled from up to 20 study centers located in Europe and up to 20 study centers located in the USA.

6.2 Inclusion Criteria

Patients must meet all of the following criteria to be considered eligible to participate in the study:

1) CF-associated lung disease with documented history of chronic infection with Gram-negative organism(s)

2) Established patient of the Principal Investigator's CF Multi-Disciplinary Team (MDT)

3) Age ≥18 years

4) Weight >40 kg

5) FEV1 >30% of predicted within the 6 months prior to study exacerbation

6) At the baseline visit: experiencing a new exacerbation of CF-associated lung disease (based on Investigator assessment of ≥4 symptoms present on the Fuchs' criteria) requiring treatment that includes an aminoglycoside antibiotic (see Section 6.4)

7) Females of childbearing potential will be included if they are either sexually inactive (sexually abstinent for 14 days prior to the first study drug dose continuing through 28 days after the last study drug dose, or using one of the following highly effective contraceptive (i.e. results in <1% failure rate when used consistently and correctly) methods in this trial:
    g. intrauterine device (IUD));
    h. surgical sterilization of the partner (vasectomy for 6 months minimum);
    i. combined (estrogen or progestogen containing) hormonal contraception associated with the inhibition of ovulation (either oral, intravaginal, or transdermal);
    j. progestogen only hormonal contraception associated with the inhibition of ovulation (either oral, injectable, or implantable);
    k. intrauterine hormone releasing system (IUS);
    l. bilateral tubal occlusion.

Sexual abstinence is considered a highly effective method only if defined as refraining from heterosexual intercourse during the entire period of risk associated with the study treatments. In this trial abstinence is only acceptable if in line with the subjects preferred and usual lifestyle.

Periodic abstinence (calendar, symptothermal, post-ovulation methods), withdrawal (coitus interruptus), spermicides only, and lactational amenorrhoea method (LAM) are not acceptable methods of contraception. As well, female condom and male condom should not be used together.

8) Females of childbearing potential agree to remain sexually inactive or to keep the same birth control method for at least 28 days following the last dose.
9) A female of non-childbearing potential must have undergone one of the following sterilization procedures at least 6 months prior to the first study drug dose:

    e. hysteroscopic sterilization;

    f. bilateral tubal ligation or bilateral salpingectomy;

    g. hysterectomy;

    h. bilateral oophorectomy;

    or be postmenopausal with amenorrhea for at least 1 year prior to the first study drug dose and follicle stimulating hormone (FSH) serum levels consistent with postmenopausal status.

10) A non-vasectomized male subject agrees to use a condom with spermicide or abstain from sexual intercourse during the study until 90 days beyond the last dose of study medication and the female partner agrees to comply with inclusion 7 or 9. For a vasectomized male who has had his vasectomy 6 months or more prior to study start, it is required that they use a condom during sexual intercourse. A male who has been vasectomized less than 6 months prior to study start must follow the same restrictions as a non-vasectomized male.

    Periodic abstinence (calendar, symptothermal, post-ovulation methods), withdrawal (coitus interruptus), spermicides only, and lactational amenorrhoea method (LAM) are not acceptable methods of contraception. As well, female condom and male condom should not be used together.

11) If male, agrees not to donate sperm from the first study drug dose until 90 days after dosing.

12) Willing and able to comply with all protocol requirements and procedures, including induction of sputum if necessary

13) Willing and able to provide signed and dated informed consent

6.3 Exclusion Criteria

Patients who meet any of the following criteria will be excluded from participation in the study:

1) Hypersensitive to cysteamine or to any of the excipients

2) Hypersensitive to penicillamine

3) Transplant recipient

4) Participation in any other interventional clinical research study (participation in observational studies is not exclusionary) within 30 days of Baseline (Day 0), and any planned participation in an interventional clinical research study for the duration of this study

5) If female, pregnancy, planned pregnancy, or breast-feeding

6) Any other significant disease/disorder which, in the Investigator's opinion, either puts the patient at risk due to study participation, or may influence the results of the study or the patient's ability to participate in the study

6.4 Fuchs' Criteria

Fuchs' Criteria is a standardized method for determining when a patient with CF is experiencing a CF exacerbation of respiratory symptoms (Fuchs et al, 1994). Based on Fuchs' Criteria an exacerbation will be deemed present if the patient has requires that the patient be treated with parenteral antibiotics for any 4 or more of the following 12 signs or symptoms:

- Change in sputum
- New or increased hemoptysis
- Increased cough
- Increased dyspnea
- Malaise, fatigue or lethargy
- Temperature above 38°C/100.4°F
- Anorexia or weight loss
- Sinus pain or tenderness
- Change in sinus discharge
- Change in physical examination of the chest

- Decrease in pulmonary function by 10% or more from a previously recorded value
- Radiographic changes indicative of pulmonary infection

Investigators will evaluate Fuchs' criteria to determine study eligibility. Patients must have ≥4 symptoms at Baseline (Day 0) for randomization into the study.

Please note that the parenteral antibiotics referenced above is only to describe the origin of Fuchs' criteria and not to suggest that subjects must be treated with parenteral antibiotics. Subjects may be treated with parenteral antibiotics while on the study.

6.5 Deviation from Inclusion/Exclusion Criteria

Deviations from the inclusion/exclusion criteria of the protocol will not be prospectively granted. If the Investigator learns that a patient who did not meet protocol eligibility criteria was entered into the study, the Investigator must immediately inform the Medical Monitor of the protocol violation so a determination can be made regarding the patient's continued participation in the study.

7 STUDY CONDUCT

It is not allowed for patients to participate in the study more than once. Participation is in this case defined as having received at least 1 dose of study medication.

7.1 General Instructions

All patients screened will be assigned a unique patient number after the informed consent form is signed. The patient number and patient initials will identify each patient throughout the study. Patient initials will comply with local requirements regarding patient confidentiality in applicable countries (e.g., AAA, BBB).

7.2 Study Procedures by Time Point

7.2.1 Screening

Screening will begin with the signing of the informed consent form. At Screening, patients will receive an explanation of the purpose and nature of the study, and will be asked to review and sign the informed consent form prior to any study-related procedures. The Screening visit may be combined with the Baseline (Day 0) visit. In the event the visits are performed on the same day, the following procedures will only be performed once: assessment of eligibility criteria, weight, and vital signs.

The following Screening procedures will be performed after signing of the informed consent:

- Document relevant medical/surgical history, including the patient's demographic information, CF diagnosis, and concurrent medical conditions.

- Review all concomitant medications (i.e., prescription, over-the-counter, supplements) and any non-medication therapies used in the 30 days prior to signing the informed consent form.

- Obtain vital signs (blood pressure, heart rate, respiratory rate, and temperature), including height and weight.

- Review inclusion/exclusion criteria, with the exception of evaluating the Fuchs' criteria if Screening and Baseline are not performed on the same day.

7.2.1.1 Screening Failures

Any patient who does not meet the entry criteria or withdraws consent prior to randomization on Day 0 will be considered a screening failure. The reason for each screen failure will be documented. Patients who screen fail may be re-screened with approval from the Medical Monitor.

7.2.2 Treatment Period

During the treatment period, patient assessments may be performed as an inpatient, an outpatient, or a combination of inpatient and outpatient assessments or at another preferred location (at visits Day 7 and Day 14 only), in accordance with the Investigator's standard of care procedures.

7.2.2.1 Baseline (Day 0)

The procedures noted below will be performed at Baseline (Day 0). In the event the Screening visit is performed on the same day as the Baseline (Day 0) visit, procedures performed for Screening do not need to be repeated.

- Obtain vital signs (blood pressure, heart rate, respiratory rate, and temperature), including weight.

- Perform a complete physical examination.

- Review any changes in concomitant medications and non-medication therapies.

- Perform spirometry to obtain the FEV1.

- Evaluate Fuchs' criteria.

- Review inclusion/exclusion criteria. If the patient continues to be eligible for study entry, randomize the patient to treatment by entering the assigned randomization number in the eCRF.

- Obtain sputum sample(s).
- Obtain blood and urine specimens for central laboratory assessments.
- Perform a urine pregnancy test (females of childbearing potential only).
- Complete the following health-related questionnaires/PROMs in the noted order: CFRSD–CRISS, Jarad and Sequeiros Symptom Score Questionnaire, and CFQ-R.
- Instruct the patient in administration of study drug. Subjects should take their first dose of study drug as close together in time as possible with their first dose of aminoglycosides. The first dose of study drug should be administered within 24 hours of the first dose of aminoglycoside.
- If the patient will continue in the study on an outpatient basis or at another preferred location (at visits Day 7 and Day 14 only), dispense study drug to the patient.
- Review AEs following administration of the first dose.

7.2.2.2 Day 7 and Day 14

The following procedures to assess CF exacerbation will be performed on Day 7 (±1 day) and Day 14 (±1 day):

- Obtain sputum sample(s).
- Obtain blood and urine specimens for laboratory assessments.
- Review any changes in concomitant medications, non-medication therapies, and document any AEs.
- Perform spirometry to obtain the FEV1.
- Obtain vital signs (blood pressure, heart rate, respiratory rate, and temperature) and weight.
- Complete the following health-related questionnaires/PROMs in the noted order: CFRSD–CRISS and Jarad and Sequeiros Symptom Score Questionnaire. The CFQ-R will only be completed on Day 14 and the Patient Global Assessment of Exacerbation will be completed following the CFQ-R.

If the patient is seen on an outpatient basis or at another preferred location (at visits Day 7 and Day 14 only), study personnel will review returned study drug inventory and reconcile any dosing discrepancies with the patient. If the patient is seen on an inpatient basis, dosing discrepancies will be assessed on a daily basis. If the patient will continue in the study on an outpatient basis, unused study drug will be returned to the patient (Day 7 only).

7.2.3 Follow-Up

7.2.3.1 Day 21 (End of Study)

The follow-up visit will be conducted at Day 21 (±1 day), or as the End of Study visit for patients who discontinue from the study prior to Day 21. The following procedures to assess CF exacerbation will be performed:

- Obtain sputum sample(s).
- Obtain blood and urine specimens for central laboratory assessments.
- Perform a urine pregnancy test (females of childbearing potential only).
- Review any changes in concomitant medications, non-medication therapies, and document any AEs.
- Perform spirometry to obtain the FEV1.
- Obtain vital signs (blood pressure, heart rate, respiratory rate, and temperature), including weight.
- Perform a complete physical examination.
- Complete the following health-related questionnaires/PROMs in the noted order: CFRSD–CRISS and Jarad and Sequeiros Symptom Score Questionnaire.

7.2.4 Unscheduled Visits

An unscheduled visit may be performed at any time during the study at the patient's request, or as deemed necessary by the Investigator. The date and reason for the unscheduled visit, as well as the procedures performed, will be documented.

7.3 Premature Discontinuation

Patients may withdraw from the study at any time. However, patients who wish to withdraw will be encouraged to undergo End of Study procedures (see Section 7.2.3.1) before withdrawing from the study. The reasons for withdrawal from the study and reason for withdrawal from study drug will be documented. Reasons for withdrawal from the study may include, but are not limited to the following:

A. Significant patient noncompliance, defined as inability or unwillingness to complete the procedures defined in the Schedule of Assessments (Section 4.3), missed/late study visits, and/or missing 6 or more consecutive doses of study drug;

B. Patient lost to follow-up (i.e., staff unable to contact patient after several attempts);

C. Request of the patient or Investigator;

D. Laboratory test results; or

E. Development of any AE, SAE, condition, intercurrent illness, injury, medical condition, or use of a medication that is likely to interfere with patient safety, the overall assessment, or the study procedures.

If a patient withdraws from the study with a clinically significant laboratory abnormality or AE, every effort must be made to follow the event until satisfactory resolution, or until the Investigator feels the event is stable and chronic in nature.

Patients who discontinue study drug prior to completing the full 14 day treatment period, if willing, should complete the remaining study visits through Day 21.

Patients enrolled who are later determined not to have experienced an exacerbation of CF-associated lung disease due to a Gram-negative organism(s) at baseline will not be discontinued from the study but will be replaced. Patients who discontinue from the study for any other reason will also be replaced. These patients will be excluded from the Per Protocol analysis.

In addition, the Sponsor reserves the right to discontinue this clinical study at any time for any reason. Such a termination must be implemented by the Investigator in a time frame that is compatible with patient well-being.

8 STUDY DRUG

Patients will be randomly assigned to one of the following treatment groups (total daily dose) in a 1:1:1:1:1:1 ratio:

- Group A: cysteamine 450 mg/day taken as one dose
- Group B: cysteamine 900 mg/day taken as two doses
- Group C: cysteamine 1,350 mg/day taken as three doses
- Group D: placebo
- Group E: cysteamine 450 mg/day taken as three doses
- Group F: cysteamine 900 mg/day taken as three doses

In order to maintain the study blind, placebo capsules will be taken so that each patient takes 3 capsules 3 times per day (i.e., morning, mid-day, and evening doses); see Table 1.

8.1 Cysteamine & Placebo Descriptions

Cysteamine (as mercaptamine bitartrate) will be provided as 150 mg hard capsules for oral administration.

To fit the blindness requirement, placebo is manufactured with common bulking excipients for immediate release formulation. Placebo will be provided as matching hard capsules.

8.2 Packaging, Shipment, and Storage

The study drug, provided by the Sponsor, will be packaged and labeled in accordance with the principles and the detailed guidelines of GMP for Medicinal Products.

In order to maintain the blind, all study drug will be identified with the following limited information: protocol number, dosing time, randomization code, administration instructions, storage requirements, and a cautionary statement according to local regulations.

Study drug will be provided to the investigational sites as capsules in blister packs. For each randomized patient, each investigator will be provided with a kit containing 2 boxes, one for every week of treatment. Each box will contain 8 daily blisters for seven days of treatment plus one day as reserve. Each daily blister will contain 9 capsules of cysteamine 150 mg or placebo. Study drug must be stored in a locked, secured, and temperature controlled storage facility accessible only to those individuals authorized by the Investigator to dispense the study drug. Study drug must be stored at room temperature (≤25°C) and prevented from freezing. Additionally, study drug must be protected from light and moisture. Patients must also be instructed on the proper storage of the product.

8.3 Dose and Administration

Administration of study drug will occur three times daily in approximately equally spaced doses (i.e., morning, mid-day, and evening); see Table 1. Study drug should be swallowed. Each dose should be taken with food or just after food, whenever possible. If food cannot be taken, then the patient can take the study drug on an empty stomach. In the event a dose is missed during the study day, the dose should be skipped (i.e., the patient should not make up the dose).

In the event the patient experiences nausea possibly due to the study drug, the investigator may prescribe a proton pump inhibitor. No dosing regimen changes will be allowed. In the event a patient is unable to tolerate the assigned dose, the patient will be discontinued from the study drug and will be requested to remain in the study until the planned completion.

8.4 Accountability and Compliance

Upon receipt of the study drug supplies, it is the responsibility of the Investigator to ensure that records of inventory and accountability are maintained. Inventory and records must be readily available for inspection by the Sponsor or its representatives and regulatory authorities at any time. For patients seen on an outpatient basis, study drug containers and any unused study drug must be returned to the investigational site by the patient at each visit for reconciliation by the Investigator, or designee.

Compliance will be assessed by a review of the inventory returned from patients treated on an outpatient basis at each visit, and on a daily basis for patients treated on

an inpatient basis. Patients will be questioned regarding any discrepancies from expected dosing.

9 DESCRIPTION OF STUDY PROCEDURES

Specific study procedures are described below. Assessment time points are provided in the Schedule of Assessments (Section 4.3). Efficacy will be assessed by sputum microbial load, sputum IL8 and neutrophil elastase levels, FEV1, weight, CRP, blood leukocyte count, assessment of blood and sputum cysteamine levels, and the following health-related questionnaires/PROMs: CFRSD-CRISS, Jarad and Sequeiros Symptom Score Questionnaire, CFQ-R, and Patient Global Assessment of Exacerbation. Safety will be assessed by collection and review of AEs, physical examinations, vital signs, and laboratory parameters (chemistry, hematology, and urinalysis).

9.1 Sputum Analysis

Quantitative and standard clinical microbiology will be performed on each sputum sample by the central lab. Results will indicate (a) total CFU count per ml and per mg and (b) organisms as Gram-positive/negative per ml and per mg.

Quantitative IL8 neutrophil and elastase assays will be performed on each sputum sample by the sponsor.

Sites may collect separate samples for each required sputum assessment or may collect one sputum sample and split it into either 2 or 3 parts (depending on sample requirements for the visit). The sample will either be split into 2 parts for Day 0, Day 7 and Day 21, and 3 parts for Day 14 or 2 samples will be collected for Day 0, Day 7 and Day 21, or 3 samples will be collected for Day 14. Each part or sample will be placed into a separate container for shipping and analyses, details. Details on sample collection, preparation and shipping are included in the Laboratory Manual.

Because the results of these analyses are potentially unblinding, they will not be made available to the investigational sites prior to database lock.

9.2 FEV1

FEV1 is the volume exhaled during the first second of a forced expiratory maneuver started from the level of total lung capacity. Three satisfactory maneuvers will be performed and the largest value of the three maneuvers will be reported. Each maneuver will be performed in accordance with American Thoracic Society (ATS)/European Respiratory Society (ERS) standards (Miller et al, 2005). For consistency, patients should be tested using the same equipment at each visit. Additionally, whenever possible, the same staff member should perform all testing for a given patient at each required visit. Patients must have a documented history of FEV1>30% of predicted within the 6 months prior to the study exacerbation.

9.3 Health-related Questionnaires/Patient-Reported Outcome Measures (PROMs)

Patients will complete the health-related questionnaires/PROMs. The questionnaires may be updated/translated in accordance with local requirements. In the event the patient is unable to complete the questionnaires without assistance, the questionnaires may be verbally answered and the patient's responses recorded by site personnel or a chaperone.

9.3.1 Cystic Fibrosis Respiratory Symptom Diary (CFRSD)–Chronic Respiratory Infection Symptom Scale (CRISS)

The CFRSD is a 16-item PROM to evaluate the effect of treatment on the severity of CF symptoms (ie, CFRSD-CRISS) and to assess the emotional and activity impacts of these symptoms. The CFRSD-CRISS is a validated unidimensional scale based on a subset of 8 items from the CFRSD questionnaire and is designed to evaluate the effect of treatment on the severity of symptoms of respiratory infection in patients with CF and a chronic respiratory infection. The 8 CFRSD-CRISS items are scored using a 5-point Likert scale ranging from 0 (no symptom) to 4 (the highest magnitude of severity). A copy of the CFRSD is provided in Section 22.1.

9.3.2 Jarad and Sequeiros Symptom Score Questionnaire

The Jarad and Sequeiros Symptom Questionnaire (Jarad and Sequieros 2011) is a simple patient-completed questionnaire that assesses and evaluates change in patient symptoms related to different aspects of respiratory function during a CF exacerbation. The questionnaire consists of 4 questions, each answered on a 4-point scale ranging from 1 (best) to 4 (worst). Individual symptom scores are summed with a total score ranging from 4 to 16. A copy of the Jarad and Sequeiros Symptom Score Questionnaire is provided in Section 22.2.

9.3.3 Cystic Fibrosis Questionnaire – Revised (CFQ-R)

The CFQ-R is a patient-completed questionnaire to assess the impact of CF and treatment on everyday life. The questionnaire consists of the following four sections: Demographics, Quality of Life, School, Work or Daily Activities, and Symptom Difficulties. A copy of the CFQ-R is provided in Section 22.3.

9.3.4 Patient Global Assessment of Exacerbation

The Patient Global Assessment of Exacerbation is a patient-completed questionnaire that assesses the current CF exacerbation in comparison to the previous exacerbation. The questionnaire consists of 6 questions, each answered on a 6-point scale, as well as 2 questions regarding the time to recovery. A copy of the Patient Global Assessment of Exacerbation is provided in Section 22.4.

9.4 Physical Examinations

A complete physical examination will be performed and will include an assessment of the following body systems: general appearance; mental status; head, eyes, ears, nose, and throat; dermatologic; cardiovascular; respiratory; gastrointestinal; and

neurological. Additional body systems may be examined at the Investigator's discretion. After the Baseline (Day 0) physical examination, any physical examination finding, with the exception of CF progression, that is assessed by the Investigator to be a clinically significant change (worsening) compared to the Baseline (Day 0) assessment, will be considered an AE and reported as described in Section 11.

9.5 Vital Signs, Height, and Weight

Vital signs include temperature and blood pressure (systolic and diastolic), heart rate, and respiratory rate assessed after 5 minutes of rest in a supine position. Weight will be measured using a calibrated scale with the patient lightly clothed and shoes off. Height may be obtained from the patient's medical history, or measured using a calibrated wall mounted stadiometer. Any vital sign or weight measurement that is assessed by the Investigator as a clinically significant change (worsening) compared to the Baseline (Day 0) assessment will be considered an AE and reported as described in Section 11.

9.6 Laboratory Testing

A central laboratory will perform all laboratory testing, with the exception of the urine pregnancy tests. The urine pregnancy test will be performed by study personnel using a pregnancy testing kit provided by the central laboratory. The procedures for laboratory collection and processing will be detailed in a separate laboratory manual.

9.6.1 Clinical Laboratory Tests

The clinical laboratory assessments to be conducted are the following:

CHEMISTRY	HEMATOLOGY	URINALYSIS (dipstick)	PREGNANCY	Sputum analysis
• CRP<br>• alkaline phosphatase<br>• aspartate aminotransferase<br>• alanine aminotransferase<br>• gamma-glutamyl transpeptidase<br>• lactate dehydrogenase<br>• total bilirubin<br>• total protein<br>• albumin<br>• blood urea nitrogen<br>• creatinine<br>• sodium<br>• phosphate<br>• potassium<br>• chloride<br>• calcium<br>• cysteamine*	• hemoglobin<br>• hematocrit<br>• mean corpuscular hemoglobin<br>• mean corpuscular hemoglobin concentration<br>• mean corpuscular volume<br>• red blood cell count<br>• platelet count<br>• white blood cell count and differential count<br>○ neutrophils<br>○ lymphocytes<br>○ eosinophils<br>○ monocytes<br>○ basophils<br>○ large unstained cells	• protein<br>• glucose<br>• ketones<br>• pH<br>• specific gravity<br>• Blood<br>• Nitrite<br>• Leukocytes<br>• Bilirubin<br>• Urobilinogen	• Urine pregnancy testing will be performed on all females of childbearing potential. In the event pregnancy is suspected, a serum pregnancy test will be performed.	• microbiology<br>• IL8 neutrophil and elastase levels<br>• cysteamine*

*cysteamine level results will only be shared once the database is locked to ensure the study is not unblinded

Reports containing laboratory safety test results will be generated by the central laboratory and transmitted to the investigational sites. In the event any clinically significant abnormal laboratory test results are identified, follow-up laboratory tests may be conducted.

Other clinical laboratory tests may be performed locally by the Investigator, as deemed necessary, to ensure patient safety.

9.6.2 Cysteamine plasma and sputum level sampling

At Day 14, a blood sample will be taken for the analysis of cysteamine levels in plasma. The cysteamine sputum level will be measured in the sample that is collected for the microbial analysis and IL8 and neutrophil elastase levels analysis. The exact time of sampling will be recorded in the eCRF, as will the time and date of the last dose taken prior to the sampling.

Because the results of these analyses are potentially unblinding, they will not be made available to the investigational sites prior to database lock.

10 CONCOMITANT MEDICATIONS, NON-MEDICATION THERAPIES, AND STUDY RESTRICTIONS

All medications (i.e., prescription, over-the-counter, supplements) taken by the patient from 30 days prior to the Screening visit and throughout the study will be documented. Documentation will include new medications, existing medications, and dose changes to existing medications. The following will be collected for all concomitant medications: medication name, start/stop dates, total daily dose, route of administration, and indication for use.

In addition, any non-medication therapies used by the patient from 30 days prior to the Screening visit and throughout the study will be documented. Documentation will include new therapies, existing therapies, and changes to existing therapies. The following will be collected for all non-medication therapies: therapy name, start/stop dates, frequency of use, and indication for use.

10.1 Prohibited Foods and Medications

10.1.1 Restricted Foods

There are no food and beverage restrictions in this study.

10.1.2 Restricted Medications

Patients will be permitted to take all of their normal medications required as part of their management of CF.

.

11 ADVERSE EVENTS

11.1 Definition of Adverse Events

An AE is defined as any untoward medical occurrence in a patient in a clinical investigation who is administered a pharmaceutical product. The AE does not necessarily have to have a causal relationship with this product. An AE can therefore be any unfavorable and unintended sign (including an abnormal laboratory finding), symptom, or disease temporally associated with the use of the investigational product, whether or not related to the investigational product.

An AE includes:

- Any symptom not previously reported in the patient’s medical history;
- A worsening of a pre-existing condition;
- A significant increase in frequency or intensity of a pre-existing condition; and
- A condition first detected or diagnosed after study drug administration, even though the condition may have been present before the start of the trial.

An AE does not include:

- Symptoms of the underlying disease (CF) that might be reasonably anticipated to come and go, or progress, given the nature and severity of the condition. However, if the progression of the disease results in hospitalization for reasons other than the exacerbation under the current study, is life-threatening, or is fatal, then progression of the disease should be reported as an SAE (see Section 12.1);

- Expected variations in severity of disease signs and symptoms that have previously been reported in the patient's medical history;

- Pre-planned medical or surgical procedures (e.g., surgery, tooth extraction, or transfusion) [Note: The condition that leads to the procedure may be an AE];

- Overdose of study drug without any clinical signs or symptoms; or

- Clinically significant laboratory values. If abnormal laboratory values are accompanied by abnormal signs or symptoms, the signs or symptoms are considered an AE and should be recorded as such. Abnormal laboratory values associated with the current CF exacerbation are not an AE. Abnormal laboratory values will be recorded in the study database.

It is the responsibility of the Investigator to document all AEs that occur after the first dose of study drug is administered, regardless of whether or not they are considered to be related to the study drug.

11.2 Documenting Adverse Events

AEs will be elicited by asking each patient a general, non-directed question such as "How have you been feeling since the last visit?" Directed questioning and examination will then be done, as appropriate. All reported AEs will be documented.

Whenever feasible, AEs should be documented as medical diagnoses (highest possible level of integration). When this is not possible, the AE should be documented in terms of the signs and/or symptoms observed by the Investigator or reported by the patient.

Information documented will include the description of the AE, the date of onset and resolution (if applicable), severity, seriousness, relationship to study procedures, action taken, and the outcome. The Investigator must follow AEs until the Day 21/End of Study visit, the event resolves, or until the condition stabilizes and the Investigator feels no further medical follow-up is warranted.

11.3 Assessment of Severity

The Investigator will categorize the **severity** of each AE according to the definitions listed below:

Mild: An event that is easily tolerated by the patient, causing minimal discomfort, and not interfering with everyday activities.

Moderate: An event that is sufficiently discomforting to interfere with normal everyday activities.

Severe: An event that prevents normal everyday activities.

Note: the term ‘severe’, used to describe the intensity, should not be confused with ‘serious’ which is a regulatory definition based on patient/event outcome or action criteria. For example, a headache may be severe but not serious, while a minor stroke is serious but may not be severe.

11.4 Assessment of Causality

After careful medical consideration, the Investigator will assess the **relationship** of the AE to the study drug according to the following categories:

Unrelated: The event is not considered to be related to the study drug.

Possibly Related: Although a relationship to the study drug cannot be completely ruled out, the nature of the event, the underlying disease, concomitant medication or temporal relationship make other explanations possible.

Probably Related: The temporal relationship and absence of a more likely explanation suggest the event could be related to the study drug.

Definitely Related: The known effects of the study drug or its therapeutic class, or based on challenge testing, suggest that study drug is the most likely cause.

All AEs/SAEs judged as having a reasonable suspected causal relationship (e.g. possibly, probably, definitely) to the study drug will be considered as ARs/serious adverse reactions (SARs).

Alternative causes such as natural history of the underlying disease, concomitant therapy, other risk factors, and the temporal relationship of the event to the treatment should be considered.

11.5 Adverse Event Outcome

The Investigator will categorize the outcome of each AE according to the following definitions:

Resolved: The patient recovered from the AE. Record the AE stop date.

Resolved with Sequelae The patient recovered from the AE with sequelae. Record the AE stop date as the date the patient recovered with sequelae.

Ongoing:	At the time of the last assessment, the event is ongoing, with an undetermined outcome. Note: ongoing AEs are not considered resolved as a result of death. No AE stop date should be recorded when an AE is ongoing.
Chronic/Stable:	At the time of last assessment, the event is ongoing and stabilized, with no change to the event outcome anticipated. Record the AE stop date.
Death:	The AE directly caused death. Record the date of death as the AE stop date.
Unknown:	There is an inability to access the patient or the patient's records to determine the outcome (e.g., patient withdraws consent or is lost to follow up). No AE stop date should be recorded.

The Investigator must follow AEs until the Day 21/End of Study visit, the event resolves, or until the condition stabilizes and the Investigator feels no further medical follow-up is warranted.

11.5.1 Withdrawal Due to Adverse Event

If a patient is withdrawn from the study by the Investigator wholly or in part due to an AE, the patient will be monitored at the discretion of the Investigator (e.g., until the event has resolved or stabilized, until the patient is referred to the care of a health care professional, or until a determination of a cause unrelated to the study drug is determined).

12 SERIOUS ADVERSE EVENT

12.1 Definition of Serious Adverse Event

A SAE is any event that meets any of the following criteria:

- Death;
- Life-threatening;
- Inpatient hospitalization or prolongation of existing hospitalization; Hospitalizations scheduled for an elective procedure or for treatment of a pre-existing condition that has not worsened during participation in the study will not be considered an SAE;
- Persistent or significant disability/incapacity;
- Congenital anomaly/birth defect in the offspring of a patient; and/or
- Other: Important medical events that may not result in death, be life-threatening, or require hospitalization, may be considered an SAE when, based

upon appropriate medical judgment, they may jeopardize the patient and may require medical or surgical intervention to prevent one of the outcomes listed in this definition. Examples of such events are:

- Intensive treatment in an emergency room or at home for allergic bronchospasm;
- Blood dyscrasias or convulsions that do not result in inpatient hospitalization; and/or
- Development of drug dependency or drug abuse.

An AE that does not meet any of the criteria for seriousness listed above will be regarded as a non-serious AE.

Definition of Terms

Life threatening: An AE is life threatening if the patient was at immediate risk of death from the event as it occurred; i.e., it does not include a reaction that if it had occurred in a more serious form might have caused death. For example, drug induced hepatitis that resolved without evidence of hepatic failure would not be considered life threatening, even though drug induced hepatitis can be fatal.

Hospitalization: AEs requiring hospitalization should be considered SAEs. Hospitalization for elective surgery or routine clinical procedures that are not the result of an AE (e.g., elective surgery for a pre-existing condition that has not worsened) need not be considered AEs or SAEs. If anything untoward is reported during the procedure, that occurrence must be reported as an AE, either 'serious' or 'non-serious' according to the defined criteria.

In general, hospitalization signifies that the patient has been detained (usually involving at least an overnight stay) at the hospital or in the emergency department for observation and/or treatment that would not have been appropriate in the physician's office or outpatient setting.

As noted in Section 11.1, if progression of the symptoms of the underlying disease (CF) result in hospitalization for reasons other than the exacerbation under the current study, if it is life-threatening, or is fatal, then progression of the disease will be reported as an SAE. If the event does not meet these criteria, then the hospitalization for the CF exacerbation under study will not be reported as an SAE.

Disability/incapacitating: An AE is incapacitating or disabling if the experience results in a substantial and/or permanent disruption of the patient's ability to carry out normal life functions.

12.2 SAE Expectedness

An SAE that is not included in the Investigator’s Brochures by its specificity, severity, outcome, or frequency is considered an unexpected AE. If a serious unexpected AE is believed to be related to the study drug or study procedures, the Sponsor will take appropriate steps to notify all Investigators participating in

sponsored clinical studies of cysteamine, as well as the appropriate regulatory authorities.

12.3 Reporting Serious Adverse Events

Any SAE that occurs at any time following the first dose of study drug or within 4 weeks following the last dose of study drug, whether or not related to the study drug, must be reported by the Investigator to the Medical Monitor within 24 hours of knowledge of the event. The Investigator should not wait to receive additional information to fully document the event before completing the initial SAE form.

Where applicable, information from relevant hospital records and autopsy reports should be obtained. In the event of a fatal or life-threatening SAE, any required follow-up must be provided within seven calendar days of the initial report. The Investigator will follow all SAEs until resolved or deemed stable and chronic in nature. Any follow-up information relevant to the SAE must be reported to the Medical Monitor.

Instances of death, cancer, or congenital abnormality, if brought to the attention of the Investigator **AT ANY TIME** after study drug administration **AND** considered by the Principal Investigator to be **RELATED TO THE STUDY DRUG**, must be reported to the Medical Monitor.

SAEs will be reported to the Sponsor according to the procedures outlined in the Study Handbook. Where required by local regulations, the Institutional Review Board/Ethics Committee (IRB/EC) and/or regulatory authorities will be informed of SAEs in accordance with local regulation timeframes and reporting requirements (as specified in the Study Handbook).

No protocol-defined AEs for expedited reporting are identified for this study.

12.3.1 Overdose

Any instance of overdose (suspected or confirmed and irrespective of whether or not the study drug was involved) must be communicated to the Sponsor in accordance with the procedures outlined in the Study Handbook.

12.3.2 Pregnancy

Pregnancy is not considered an AE or SAE; however, the Investigator must collect pregnancy information for female patients or female partners of male patients who become pregnant while participating in a study. The Investigator should report the pregnancy to the Sponsor in accordance with the procedures outlined in the Study Handbook.

Any pregnancy that occurs in a patient or patient’s partner during a study should be followed to outcome. In some circumstances, it may be necessary to monitor the development of the newborn for an appropriate period post-delivery.

Should the patient or patient’s partner not wish for the pregnancy to be followed to outcome or beyond, this should be documented.

Female patients who become pregnant will be advised to discontinue study drug immediately, and will not be administered further study drug.

13 STATISTICS

A comprehensive statistical analysis plan (SAP) will be developed for this study and finalized prior to locking the database. The SAP will provide additional details about the specific planned analyses and hypothesis tests.

13.1 Analysis Populations

The analysis population for all efficacy and safety analyses will include all patients who received at least one dose of study drug, which will be referred to as the Safety Population.

The Per Protocol (PP) population will include all randomized patients who complete the Treatment Period without experiencing a significant protocol violation that would impact critical safety and efficacy interpretation (eg, missing 6 or more consecutive doses, determined not to have experienced an exacerbation of CF-associated lung disease due to a Gram-negative organism(s) at baseline).

13.2 General Procedures

All analyses will be performed and all tables, figures and data listings will be prepared using SAS, Version 9.4 or higher.

Continuous variables will be summarized descriptively by the number of patients with non-missing data, mean and standard deviation, median, minimum, and maximum values.

Categorical variables will be summarized descriptively by their counts and associated percentages.

13.3 Sample Size

A total of approximately 120 patients with CF being treated for an exacerbation of CF-associated lung disease will be enrolled in the study: 20 patients in each group. The sample size of approximately 120 patients was selected empirically without a formal statistical assumption. The sample size selection is considered to be appropriate to determine the optimal dose and regimen based on evidence of efficacy and acceptable safety and tolerability profile as well as establish point estimates and variability for efficacy endpoints for future evaluation.

A sample size of 20 patients in each group will have approximately 80% power to detect a 1.2 log reduction in sputum bacterial load from baseline over placebo, assuming a standard deviation of 1.31 in the reduction of sputum bacterial load, based on a two-sided, two-sample t-test at the 5% level of significance. This estimated

standard deviation is that reported in a 2-week study of CF patients with Pseudomonas who were treated during exacerbations with 2 weeks of intravenous tobramycin (Al-Aloul et al, 2014).

13.4 Statistical Methods

13.4.1 Demographic and Baseline Characteristics

Demographics and baseline characteristics will be summarized using descriptive statistics.

13.4.2 Efficacy Analyses

The primary objective of this study, to determine the optimal dose and regimen of cysteamine in patients with exacerbations of CF-associated lung disease, will be investigated through a combination of safety, efficacy, and microbiology endpoints. Though statistical comparisons between treatment groups with respect to efficacy and microbiology will be performed to help inform the selection of the final dose for further development, safety and tolerability will also be paramount in the selection of the optimal dose.

The primary efficacy endpoint for the study is:

- Change from baseline (Day 0) in log-transformed sputum bacterial load of gram negative CFU per ml and per mg following a CF exacerbation

The secondary efficacy endpoints for the study are:

- Change from baseline (Day 0) in patient health-related questionnaires/PROMs (CFRSD-CRISS, Jarad and Sequeiros Symptom Score Questionnaire, and CFQ-R)
- Patient Global Assessment of Exacerbation outcome

- Change from baseline (Day 0) in sputum IL8 and neutrophil elastase levels following a CF exacerbation

- Change from baseline (Day 0) in the following:
  - FEV1
  - Weight and BMI
  - CRP
  - Blood leukocyte count

- Assessment of blood and sputum cysteamine levels at Day 14

The efficacy comparisons of interest at Day 14 will be conducted using a linear mixed model for repeated measures, analysis of variance (ANOVA), and chi-square tests as appropriate. Change from baseline (Day 0) to Day 7 and Day 21 will also be assessed where applicable. All analyses will be conducted using two-sided tests at the

alpha=0.05 level of significance. There will be no adjustments for multiple comparisons in the efficacy analyses for this study.

The data for change from baseline (Day 0) measures will be summarized with descriptive statistics and confidence intervals and will be analyzed via contrast statements from the mixed model from all pairwise and pooled total daily dose (TDD) group comparisons, each at the alpha=0.05 level of significance. Analyses of subgroups and other exploratory linear mixed models may be explored.

Continuous variables will be summarized descriptively by the number of patients with non-missing data, mean and standard deviation, median, minimum, and maximum values. Categorical variables will be summarized descriptively by their counts and associated percentages.

13.4.3 Safety Analyses

Safety analyses will involve the examination of the descriptive statistics and individual patient listings for any effects of study treatment on clinical safety. Summaries will be prepared by treatment group and TDD group.

Treatment-emergent adverse event (TEAE) summaries will include the overall incidence (by system organ class and preferred term), events by maximum intensity, events by relationship to study treatment, events leading to discontinuation of study drug, and SAEs.

Vital signs and laboratory parameters (hematology, chemistry, and urinalysis) will be summarized descriptively by treatment. Actual and change from baseline (Day 0) data will be calculated and summarized.

AEs and concomitant medications will be coded using standardized medical dictionaries.

13.5 Interim Analysis

There will be no interim analysis for this study.

14 ETHICS AND RESPONSIBILITIES

14.1 Good Clinical Practice

This study will be conducted in accordance with the International Conference on Harmonisation (ICH) Good Clinical Practice (GCP) Consolidated Guideline (E6) and any applicable national and local laws and regulations (e.g., Title 21 Code of Federal Regulations [21CFR] Parts 11, 50, 54, 56, 312, and 314, EU Directive 20/EC and 28/EC). Any episode of noncompliance will be documented.

The Investigator is responsible for performing the study in accordance with this protocol and the applicable GCP guidelines referenced above for collecting, recording, and accurately reporting the data.

The Investigator is responsible for ensuring the privacy, health, and welfare of the patients during and after the study, and must ensure that trained personnel are immediately available in the event of a medical emergency. The Investigator and the applicable study staff must be familiar with the requirements of the study and with the properties of the study drug described in the Investigator's Brochure.

The Investigator at each investigational site has the overall responsibility for the conduct and administration of the study at that site and for contacts with study management, the IRB/EC, and with local authorities.

14.2 Data Safety Monitoring Board (DSMB)

The DSMB will review safety data periodically throughout the study with special attention to all AEs, SAEs, and other measures of safety in order to monitor the emerging safety profile, and recommend modification or premature termination of the study, if necessary. The DSMB will be composed of independent physicians with expertise in the relevant therapeutic field, as well as other relevant experts, such as a statistician. The DSMB Charter will outline the data to be reviewed by the DSMB and the frequency of the meetings.

14.3 Institutional Review Board (IRB)/Ethics Committee (EC)

Before initiation of the study, the Investigator must submit the protocol, Investigator's Brochure, informed consent form, advertisements, and any written materials that will be made available to the patient to an IRB/EC complying with the provisions specified in the ICH guidelines for approval. Written IRB/EC approval of the noted documents must be obtained prior to initiation of the study.

The Investigator and/or Sponsor are responsible for reporting the following to the IRB/EC, if applicable, in accordance with local and IRB/EC regulations:

- All SAEs regardless of cause and whether anticipated or unanticipated (reported per IRB/EC regulations)
- Significant findings that become known during the course of the study that might affect the willingness of patients to continue to participate
- Protocol or consent amendments prior to the implementation of the change
- Study progress reports at least once a year, if applicable
- Notification of study completion or termination

Details of reporting requirements are provided in the Study Handbook.

14.4 Informed Consent

The Investigator, or designee, will thoroughly explain to the patient the purpose of the study, the associated procedures, as well as any expected effects and ARs before any study-specific screening procedures are conducted.

The Investigator will explain that the patient is completely free to refuse participation in the study or to withdraw from the study at any time and for any reason. Similarly, the Investigator and/or Sponsor will be free to withdraw the patient at any time for safety or administrative reasons. Any other requirements necessary for the protection of the human rights of the patient will also be explained according to current ICH GCP guidelines (ICH E6 1997) and the Declaration of Helsinki, 1964 (as amended in 2013); see Section 22.5.

The patient will be provided with an informed consent form and will be given sufficient time and opportunity to inquire about the details of the study and to decide whether or not to allow the patient to participate. The patient and the study personnel with whom he/she discusses the informed consent, will sign and date the consent form.

14.5 Records Management and Study Monitoring

Representatives of the Sponsor may request access to all study records for inspection throughout the study or after study completion. Such access must be stated in the informed consent form signed by the patient.

The Sponsor is responsible for ensuring the protocol conduct of the study with regard to ethics, protocol adherence and site procedures, integrity of the data, and applicable laws and/or regulations. Study monitoring will be conducted at regular intervals during the study and following completion of the study. Study monitors will contact the investigational site via visits to the site, telephone calls, and other communication methods in order to review the progress of the study. During the monitoring visits, the following aspects of the study conduct will be carefully reviewed: informed consent of patients, patient recruitment, compliance with study procedures, source data verification, AE and SAE documentation and reporting, and quality of the data. The Investigators must make all study data accessible to the clinical monitor, to other authorized representatives of the Sponsor, members of the IRB/EC, and regulatory inspectors. The informed consent form signed by the patient must indicate the possibility for review of the data by these authorized individuals/entities.

14.6 Source Documentation and Data Collection

All patient-related data will be documented in case report forms (CRFs) in a confidential fashion, with the patient identified by patient number and initials only. Study data will be collected using an electronic data capture (EDC) system.

All the information required by the protocol must be documented and any omissions explained. The Investigator must review all CRF entries for completeness and accuracy.

Source documents, including all demographic and medical information, CRFs, and informed consent form for each patient in the study must be maintained by the Investigator. All information in the CRFs must be traceable to the original source documents. Examples of source documents include hospital records, outpatient visit records, physician notes, consulting physician notes, laboratory reports, study drug inventory records, and patient dosing data. Records may be written in hard copy or in

an electronic medical records system. The Investigator must maintain the original source document records for each patient for the length of time required by the Sponsor.

14.7 Study Files and Record Retention

All data relating to the study, including the contents of the Investigator's Site Files, patient CRFs, financial records, and other source data will be stored by the Investigator until written notification is received from the Sponsor indicating that records no longer require storage.

If the Investigator cannot guarantee this archiving requirement at the study site, arrangements must be made between the Investigator and the Sponsor to store the documents in an alternative secure facility. Study documents should not be destroyed without written approval from the Sponsor.

14.8 Declaration of End of Clinical Study

For investigational sites located in the EU, a declaration of the end of the clinical study will be made according to the procedures outlined in Directive 2001/20/ED, Article 10(c); for other countries, local regulations will be followed.

14.9 Registration of Clinical Study

This clinical study will be registered on clinical trial registry websites, according to the Sponsor's standard procedures.

15 AUDITING

Sponsor representatives may audit some investigational sites to evaluate study conduct and compliance with protocols, Standard Operating Procedures (SOPs), GCPs, and applicable regulatory requirements. Additionally, regulatory authorities and IRB/EC representatives may also perform audits to verify compliance with GCP guidelines. The Investigator must permit these inspections as part of the study conduct.

16 AMENDMENTS

Protocol modifications, except those intended to reduce immediate risk to study patients, may only be made by the Sponsor. A protocol change intended to eliminate an apparent immediate hazard to patients may be implemented immediately, provided the IRB/EC is notified in accordance with their procedures.

Any permanent change to the protocol must be handled as a protocol amendment. The written amendment must be submitted to the IRB/EC, and the Investigator must receive approval before implementing the change(s).

If in the judgment of the IRB/EC, the Investigator, and/or the Sponsor, the amendment to the protocol substantially changes the study design and/or increases the potential risk to the patient and/or has an impact on the patient's involvement as a study participant, the currently approved written informed consent form will require

similar modification. In such cases, informed consent will be renewed for patients enrolled in the study before continued participation.

17 FINANCING AND INSURANCE

A separate financial agreement will be made between the Investigator and the Sponsor before study drug is shipped to the investigational site. The Investigator will provide the Sponsor with financial information required to complete USA Food and Drug Administration (FDA) Form FDA 3454, as well as any additional information required for local regulatory authorities. Each Investigator will notify the Sponsor of any relevant changes in this financial information during the conduct of the study, and for 1 year after study completion.

This clinical study is insured in accordance with the corresponding local legal provisions. The policy coverage is subject to the full policy terms, conditions, extensions, and exclusions. Excluded from the insurance coverage are damages to health, and worsening of previous existing disease that would have occurred or continued if the patient had not taken part in the clinical study. The policy regarding clinical trials insurance will be provided to the investigational sites by the Sponsor.

18 STUDY REPORT AND PUBLICATIONS

The Sponsor is responsible for preparing a clinical study report based on the results of this study. The Sponsor’s publication policy is discussed in the Investigator's Clinical Research Agreement.

19 STUDY DISCONTINUATION

Both the Sponsor and the Investigator reserve the right to terminate the study at the Investigator’s site at any time. Should this be necessary, the Investigator will inform the IRB/EC of the same. In terminating the study, the Sponsor and the Investigator will assure that adequate consideration is given to the protection of the patients’ interests.

20 CONFIDENTIALITY

All information generated in this study is available to authorized regulatory officials, IRB/EC personnel, the Sponsor and its authorized representatives are allowed full access to the records. Appropriately anonymized data relating to the results of this trial can be published under the Creative Commons Attribution (CC BY) 4.0 license

Identification of patients in CRFs shall be by initials and screening numbers only. If required by local agencies, initials will be coded per local regulations (e.g., AAA, BBB). Additionally, if required, the patient's full name may be made known to an authorized regulatory agency or other authorized official.

21 REFERENCES

Al-Aloul, M., Nazareth, D., Walshaw, M. Nebulized tobramycin in the treatment of adult CF pulmonary exacerbations. J. Aerosol Med and Pulmonary Drug Del 2014. 27 (4): 299-305.

Bals R, Hubert D, Tümmler B. Antibiotic treatment of CF lung disease: From bench to bedside. J Cystic Fibrosis 2011; 10 Suppl 2: S146–S151.

Besouw M, Masereeuw R, van den Heuvel L, Levtchenko E. Cysteamine: an old drug with new potential. Drug Discovery Today 2013, 18; 15/16: 785-92.

Britto MT, Kotagal UR, Hornung RW, et al. Impact of recent pulmonary exacerbations on quality of life in patients with cystic fibrosis. Chest 2002; 121:64–72.

Charrier C, Rodger C, Robertson J, Kowalczuk A, Shand N, Fraser-Pitt D, Mercer D, O'Neil D. Cysteamine (Lynovex®), a novel mucoactive antimicrobial & antibiofilm agent for the treatment of cystic fibrosis. Orphanet Journal of Rare Diseases 2014 9:189.

Devereux G, Fraser-Pittb, D, Robertsonb, J, Devlina, E, Mercerb, D, O'Neil, D. Cysteamine as a Future Intervention in Cystic Fibrosis Against Current and Emerging Pathogens: A Patient-based ex vivo Study Confirming its Antimicrobial and Mucoactive Potential in Sputum. EBIOM-00245 Article in Press August 2015.

Fuchs HJ, Borowitz, DS, Christiansen, DH. Effect of aerosolized recombinant human DNase on exacerbations of respiratory symptoms and on pulmonary function in patients with cystic fibrosis. The Pulmozyme Study Group. N Engl J Med 1994;331:637–642.

Goss CH, Burns JL. Exacerbations in cystic fibrosis. 1: Epidemiology and pathogenesis. Thorax 2007; 62:360–367.

Goss CH, Quittner AL. Patient-reported outcomes in cystic fibrosis. Proc Am Thorac Soc 2007; 4:378–386.

Giwercman B, Lambert PA, Rosdahl VT, Shand GH, Hoiby N. Rapid emergence of resistance in Pseudomonas aeruginosa in cystic fibrosis patients due tot in vivo selection of stable partially derepressed B-lactamase producing strains. J Antimicrobial Chemo 1990; 26: 247-59.

Hegarty M, MacDonald J, Watter P, Wilson C. Quality of life in young people with cystic fibrosis: effects of hospitalization, age and gender, and differences in parent/child perceptions. Child: Care, Health Develop 2009; 35:462–468.

Hoiby N. New antimicrobials in the management of cystic fibrosis. J Antimicrobial Chem, 2002; 49:235-238.

Jarad, NA and Sequeiros, IM. A novel respiratory symptom scoring system for CF pulmonary exacerbations. Q J Med 2012; 105:137–143.

Koch C, Pedersen S, Jensen E, et al. Retrospective clinical study of hypersensitivity reactions to aztreonam and six other beta-lactam antibiotics in cystic fibrosis patients receiving multiple treatment courses. Rev Infect Dis 1991;13:S608–11.

Miller MR et al. Standardisation of spirometry. Eur Resp J 2005;26:319-38.

Oliver A, Canton R, Campo P, Baquero F, Blazquez J. High frequency of hypermutatable Pseudomonas aeruginosa in cystic fibrosis lung infection. Science 2000; 288:1251-3.

Pitt TL, Sparrow M, Warner M, Stefanidou M. Survey of resistance of *Pseudomonas aeruginosa* from UK patients with cystic fibrosis to six commonly prescribed antimicrobial agents. *Thorax* 2003;58:794–6.

Pleasants R, Samuelson W. Allergic reactions to parenteral beta-lactam antibiotics in patients with cystic fibrosis. Chest 1994;106:1124–8.

Sanders DB, Bittner RCL, Rosenfeld M, et al. Failure to recover to baseline pulmonary function after cystic fibrosis pulmonary exacerbation. Am J Respir Crit Care Med 2010; 182:627–632.

Sanders DB, Bittner RCL, Rosenfeld M, et al. Pulmonary exacerbations are associated with subsequent FEV1 decline in both adults and children with cystic fibrosis. Pediatr Pulmonol 2011; 46:393–400.

Stewart PS, Costerton JW. Antibiotic resistance of bacteria in biofilms. Lancet 2007; 358:135-8.

Ting, Naitee(2009) 'Practical and Statistical Considerations in Designing an Early Phase II. Osteoarthritis Clinical Trial: A Case Study', Communications in Statistics - Theory and Methods, 38: 18, 3282 — 3296

UK CF Registry, Annual Data Report 2014
http://www.cysticfibrosis.org.uk/media/598466/annual-data-report-2013-jul14.pdf

Wills R, Henry RL, J Francis. Antibiotic hypersensitivity reactions in cystic fibrosis. J Paediatr Child Health 1998;43:325–9.

22 APPENDICES

22.1 APPENDIX A – Cystic Fibrosis Respiratory Symptom Diary (CFRSD) – Chronic Respiratory Infection Symptom Scale (CRISS)

Cystic Fibrosis: Your Daily Experience

Instructions:

- Complete this diary between 5:00 P.M. and when you go to bed each evening.
- Think carefully about **your experience with cystic fibrosis, specifically during the last 24 hours**, before responding to each question. The "last 24 hours" is the amount of time that has passed since the same time the previous day.
- ***Please complete all of the questions in one sitting if possible***.

What is today's date? *(write-in your answer)*:	___________, ______20____<br>MONTH DAY YEAR
What is the current time? *(write-in your answer, and circle "AM" (before noon) or "PM" (after noon):*	AM<br>_____:_____o' clock

30

During the last 24 hours…

1. How difficult was it to breathe? *(Check one)*

- Not difficult ☐
- A little difficult ☐
- Somewhat difficult ☐
- A good deal difficult ☐
- A great deal difficult ☐

During the last 24 hours…

2. How feverish did you feel (have a temperature)? *(Check one)*

- Not feverish ☐
- A little feverish ☐
- Somewhat feverish ☐
- A good deal feverish ☐
- A great deal feverish ☐

During the last 24 hours…

3. How tired did you feel? *(Check one)*

- Not tired ☐
- A little tired ☐
- Somewhat tired ☐
- A good deal tired ☐
- A great deal tired ☐

During the last 24 hours…

4. How bad were your chills or sweats? *(Check one)*

- No chills or sweats .... ☐
- Slightly Bad .... ☐
- Moderately Bad .... ☐
- Very Bad.. ☐
- Extremely Bad.. ☐

During the last 24 hours…

5. How bad was your cough? *(Check one)*

- No cough.. ☐
- Slightly Bad .... ☐
- Moderately Bad .... ☐
- Very Bad.. ☐
- Extremely Bad.. ☐

During the last 24 hours…

6. How much mucus did you cough up? *(Check one)*

- No mucus .... ☐
- A little mucus .... ☐
- Some mucus... ☐
- A good deal of mucus .... ☐
- A great deal of mucus................................... ☐

During the last 24 hours…

7. How much tightness in the chest did you have? *(Check one)*

- No tightness……………………………………………☐
- A little tightness……………………………………☐
- Some tightness ………………………………………☐
- A good deal of tightness …………………………☐
- A great deal of tightness…………………………☐

During the last 24 hours…

8. How bad was your wheezing? *(Check one)*

- No wheezing …………………………………………☐
- Slightly Bad ……………………………………………☐
- Moderately Bad ………………………………………☐
- Very Bad…………………………………………………☐
- Extremely Bad…………………………………………☐

During the last 24 hours…

9. How difficult was it to sleep? *(Check one)*

- Not difficult……………………………………………☐
- A little difficult ………………………………………☐
- Somewhat difficult…………………………………☐
- A good deal difficult………………………………☐
- A great deal difficult ……………………………☐

During the last 24 hours…

10. How worried were you about your cystic fibrosis?
(Check one)

- Not worried ☐
- A little worried ☐
- Somewhat worried ☐
- A good deal worried ☐
- A great deal worried ☐

During the last 24 hours…

11. How cranky did you feel?
(Check one)

- Not cranky ☐
- A little cranky ☐
- Somewhat cranky ☐
- A good deal cranky ☐
- A great deal cranky ☐

During the last 24 hours…

12. How sad or depressed did you feel? *(Check one)*

- Not sad or depressed ☐
- A little sad or depressed ☐
- Somewhat sad or depressed ☐
- A good deal sad or depressed ☐
- A great deal sad or depressed ☐

During the last 24 hours…

13. How frustrated did you feel? *(Check one)*

- Not frustrated ☐
- A little frustrated ☐
- Somewhat frustrated ☐
- A good deal frustrated ☐
- A great deal frustrated ☐

During the last 24 hours…

14. How much time did you spend sitting or lying down? *(Check one)*

- Hardly any of the time ☐
- Some of the time ☐
- Most of the time ☐
- All of the time ☐

During the last 24 hours…

15. Did you reduce your usual activities? *(Check one)*

- Yes ☐
- No ☐

During the last 24 hours…

16. Did you miss work or school? *(Check one)*

- Yes ☐
- No ☐
- Does not apply, I did not have *work* or school in the last 24 hours ☐

22.2 APPENDIX B – Jarad and Sequeiros Symptom Score Questionnaire

Subjectnumber: ________________ Date Completed: _ _-_ _ _-_ _ _ _

Jarad and Sequeiros Symptom Score Questionnaire

The following questions are about your current symptoms.
Please tick the box indicating your answer.

Cough

No cough	☐	1
Cough in the morning	☐	2
Cough most of the day	☐	3
Cough all day	☐	4

Sputum volume and viscosity

No sputum	☐	1
Less than one egg cupful	☐	2
One egg cupful and thick	☐	3
More than one egg cupful and thick	☐	4

Breathlessness

No breathlessness	☐	1
Breathlessness when climbing a hill	☐	2
Breathlessness walking at a level ground	☐	3
Breathlessness when walking within the house	☐	4

Fatigue

Tired at the end of the day	☐	1
Tired easily	☐	2
Tired after a minor effort	☐	3
Tired at rest	☐	4

22.3 APPENDIX C – Cystic Fibrosis Questionnaire – Revised (CFQ-R)

CFQ-R CYSTIC FIBROSIS QUESTIONNAIRE - REVISED

Adolescents and Adults (Patients 14 Years Old and Older)

Section II. Quality of Life

Please tick the box indicating your answer.

*During the past **two weeks**, to what extent have you had difficulty:*	A lot of difficulty	Some difficulty	A little difficulty	No difficulty
**1.** Performing vigorous activities such as running or playing sports	☐	☐	☐	☐
**2.** Walking as fast as others	☐	☐	☐	☐
**3.** Carrying or lifting heavy things such as books, shopping, or school bags	☐	☐	☐	☐
**4.** Climbing one flight of stairs	☐	☐	☐	☐
**5.** Climbing stairs as fast as others	☐	☐	☐	☐

***During the past two weeks**, indicate how often:*	Always	Often	Sometimes	Never
**6.** You felt well	☐	☐	☐	☐
**7.** You felt worried	☐	☐	☐	☐
**8.** You felt useless	☐	☐	☐	☐
**9.** You felt tired	☐	☐	☐	☐
**10.** You felt full of energy	☐	☐	☐	☐
**11.** You felt exhausted	☐	☐	☐	☐
**12.** You felt sad	☐	☐	☐	☐

Please circle the number indicating your answer. Please choose only one answer for each question.

*Thinking about the state of your health over the last **two weeks**:*

13. To what extent do you have difficulty walking?
1. You can walk a long time without getting tired
2. You can walk a long time but you get tired
3. You cannot walk a long time because you get tired quickly
4. You avoid walking whenever possible because it's too tiring for you

14. How do you feel about eating?
1. Just thinking about food makes you feel sick
2. You never enjoy eating
3. You are sometimes able to enjoy eating
4. You are always able to enjoy eating

15. To what extent do your treatments make your daily life more difficult?
1. Not at all
2. A little
3. Moderately
4. A lot

Cystic Fibrosis Foundation

©2000, Quittner, Modi, Watrous and Messer. Revised 2002. CFQ-R Teen/Adult, English Version 2.0
©2005, Bryon and Stramik. CFQ-R Teen/Adult, UK-English Language Version 2.0

Page 2

CFQ-R CYSTIC FIBROSIS QUESTIONNAIRE - REVISED

Adolescents and Adults (Patients 14 Years Old and Older)

16. How much time do you currently spend each day on your treatments?
1. A lot
2. Some
3. A little
4. Not very much

17. How difficult is it for you to do your treatments (including medications) each day?
1. Not at all
2. A little
3. Moderately
4. Very

18. How do you think your health is now?
1. Excellent
2. Good
3. Fair
4. Poor

Please select a box indicating your answer.

*Thinking about your health during the past **two weeks**, indicate the extent to which each sentence is true or false for you.*	Very true	Somewhat true	Somewhat false	Very false
**19.** I have trouble recovering after physical effort	☐	☐	☐	☐
**20.** I have to limit vigorous activities such as running or playing sports	☐	☐	☐	☐
**21.** I have to force myself to eat	☐	☐	☐	☐
**22.** I have to stay at home more than I want to	☐	☐	☐	☐
**23.** I feel comfortable discussing my illness with others	☐	☐	☐	☐
**24.** I think I am too thin	☐	☐	☐	☐
**25.** I think I look different from others my age	☐	☐	☐	☐
**26.** I feel bad about my physical appearance	☐	☐	☐	☐
**27.** People are afraid that I may be contagious	☐	☐	☐	☐
**28.** I get together with my friends a lot	☐	☐	☐	☐
**29.** I think my coughing bothers others	☐	☐	☐	☐
**30.** I feel comfortable going out at night	☐	☐	☐	☐
**31.** I often feel lonely	☐	☐	☐	☐
**32.** I feel healthy	☐	☐	☐	☐
**33.** It is difficult to make plans for the future (for example, going to college, getting married, getting promoted at work, etc.)	☐	☐	☐	☐
**34.** I lead a normal life	☐	☐	☐	☐

Cystic Fibrosis Foundation

©2000, Quittner, Modi, Watrous and Messer. Revised 2002. CFQ-R Teen/Adult, English Version 2.0
©2005, Bryon and Stramik. CFQ-R Teen/Adult, UK-English Language Version 2.0

Page 3

CFQ-R CYSTIC FIBROSIS QUESTIONNAIRE - REVISED

Adolescents and Adults (Patients 14 Years Old and Older)

Section III. School, Work, or Daily Activities

Questions 35 to 38 are about school, work, or other daily tasks.

35. To what extent did you have trouble keeping up with your schoolwork, professional work, or other daily activities during the past **two weeks**?
1. You have had no trouble keeping up
2. You have managed to keep up but it's been difficult
3. You have been behind
4. You have not been able to do these activities at all

36. How often were you absent from school, work, or unable to complete daily activities during the last two weeks because of your illness or treatments?
☐ Always ☐ Often ☐ Sometimes ☐ Never

37. How often does CF get in the way of meeting your school, work, or personal goals?
☐ Always ☐ Often ☐ Sometimes ☐ Never

38. How often does CF interfere with getting out of the house to run errands such as shopping or going to the bank?
☐ Always ☐ Often ☐ Sometimes ☐ Never

Section IV. Symptom Difficulties

Please select a box indicating your answer.

*Indicate how you have been feeling during the past **two weeks**.*	A great deal	Somewhat	A little	Not at all
**39.** Have you had trouble gaining weight?	☐	☐	☐	☐
**40.** Have you been congested?	☐	☐	☐	☐
**41.** Have you been coughing during the day?	☐	☐	☐	☐
**42.** Have you had to cough up mucus?	☐	☐	☐	☐ Go to Question 44

43. Has your mucus been mostly: ☐ Clear ☐ Clear to yellow ☐ Yellowish-green ☐ Green with traces of blood ☐ Don't know

*How often during the past **two weeks:***	Always	Often	Sometimes	Never
**44.** Have you been wheezing?	☐	☐	☐	☐
**45.** Have you had trouble breathing?	☐	☐	☐	☐
**46.** Have you woken up during the night because you were coughing?	☐	☐	☐	☐
**47.** Have you had problems with wind?	☐	☐	☐	☐
**48.** Have you had diarrhoea?	☐	☐	☐	☐
**49.** Have you had abdominal pain?	☐	☐	☐	☐
**50.** Have you had eating problems?	☐	☐	☐	☐

Please make sure you have answered all the questions.

THANK YOU FOR YOUR COOPERATION!

Cystic Fibrosis Foundation

©2000, Quittner, Modi, Watrous and Messer. Revised 2002. CFQ-R Teen/Adult, English Version 2.0
©2005, Bryon and Stramik. CFQ-R Teen/Adult, UK-English Language Version 2.0

Page 4

22.4 APPENDIX D – Patient Global Assessment of Exacerbation

Subject number: ________________ Date Completed: _ _-_ _ _-_ _ _ _

Patient Global Assessment of Exacerbation

1. After how many days of treatment did you start to feel better: __ __ Days
   or ☐ I don't feel any better

2. After how many days of treatment did you feel 'back to normal' __ __ Days
   or ☐ I haven't got back to normal

Comparing <u>this</u> chest infection with <u>previous</u> chest infections:

3. My cough got better more quickly:
   - Strongly Agree ☐
   - Agree ☐
   - Neutral ☐
   - Disagree ☐
   - Strongly Disagree ☐
   - I don't know ☐

4. The colour of my sputum returned to normal more quickly:
   - Strongly Agree ☐
   - Agree ☐
   - Neutral ☐
   - Disagree ☐
   - Strongly Disagree ☐
   - I don't know ☐

5. My breathing got better more quickly:
   - Strongly Agree ☐
   - Agree ☐
   - Neutral ☐
   - Disagree ☐
   - Strongly Disagree ☐
   - I don't know ☐

6. My appetite returned to normal more quickly:
   - Strongly Agree ☐
   - Agree ☐
   - Neutral ☐
   - Disagree ☐
   - Strongly Disagree ☐
   - I don't know ☐

7. My energy levels returned to normal more quickly:
   - Strongly Agree ☐
   - Agree ☐
   - Neutral ☐
   - Disagree ☐
   - Strongly Disagree ☐
   - I don't know ☐

8. My ability to return to normal activity levels happened more quickly:
   - Strongly Agree ☐
   - Agree ☐
   - Neutral ☐
   - Disagree ☐
   - Strongly Disagree ☐
   - I don't know ☐

22.5 APPENDIX E – Declaration of Helsinki

Clinical Review & Education

Special Communication

World Medical Association Declaration of Helsinki
Ethical Principles for Medical Research Involving Human Subjects

World Medical Association

Adopted by the 18th WMA General Assembly, Helsinki, Finland, June 1964, and amended by the:
29th WMA General Assembly, Tokyo, Japan, October 1975
35th WMA General Assembly, Venice, Italy, October 1983
41st WMA General Assembly, Hong Kong, September 1989
48th WMA General Assembly, Somerset West, Republic of South Africa, October 1996
52nd WMA General Assembly, Edinburgh, Scotland, October 2000
53rd WMA General Assembly, Washington, DC, USA, October 2002 (Note of Clarification added)
55th WMA General Assembly, Tokyo, Japan, October 2004 (Note of Clarification added)
59th WMA General Assembly, Seoul, Republic of Korea, October 2008
64th WMA General Assembly, Fortaleza, Brazil, October 2013

Preamble

1. The World Medical Association (WMA) has developed the Declaration of Helsinki as a statement of ethical principles for medical research involving human subjects, including research on identifiable human material and data.

   The Declaration is intended to be read as a whole and each of its constituent paragraphs should be applied with consideration of all other relevant paragraphs.

2. Consistent with the mandate of the WMA, the Declaration is addressed primarily to physicians. The WMA encourages others who are involved in medical research involving human subjects to adopt these principles.

General Principles

3. The Declaration of Geneva of the WMA binds the physician with the words, "The health of my patient will be my first consideration," and the International Code of Medical Ethics declares that, "A physician shall act in the patient's best interest when providing medical care."

4. It is the duty of the physician to promote and safeguard the health, well-being and rights of patients, including those who are involved in medical research. The physician's knowledge and conscience are dedicated to the fulfilment of this duty.

5. Medical progress is based on research that ultimately must include studies involving human subjects.

6. The primary purpose of medical research involving human subjects is to understand the causes, development and effects of diseases and improve preventive, diagnostic and therapeutic interventions (methods, procedures and treatments). Even the best proven interventions must be evaluated continually through research for their safety, effectiveness, efficiency, accessibility and quality.

7. Medical research is subject to ethical standards that promote and ensure respect for all human subjects and protect their health and rights.

8. While the primary purpose of medical research is to generate new knowledge, this goal can never take precedence over the rights and interests of individual research subjects.

9. It is the duty of physicians who are involved in medical research to protect the life, health, dignity, integrity, right to self-determination, privacy, and confidentiality of personal information of research subjects. The responsibility for the protection of research subjects must always rest with the physician or other health care professionals and never with the research subjects, even though they have given consent.

10. Physicians must consider the ethical, legal and regulatory norms and standards for research involving human subjects in their own countries as well as applicable international norms and standards. No national or international ethical, legal or regulatory requirement should reduce or eliminate any of the protections for research subjects set forth in this Declaration.

11. Medical research should be conducted in a manner that minimises possible harm to the environment.

12. Medical research involving human subjects must be conducted only by individuals with the appropriate ethics and scientific education, training and qualifications. Research on patients or healthy volunteers requires the supervision of a competent and appropriately qualified physician or other health care professional.

Copyright 2013 American Medical Association. All rights reserved.

Downloaded From: http://jama.jamanetwork.com/ by a American Medical Association User on 09/23/2015

13. Groups that are underrepresented in medical research should be provided appropriate access to participation in research.

14. Physicians who combine medical research with medical care should involve their patients in research only to the extent that this is justified by its potential preventive, diagnostic or therapeutic value and if the physician has good reason to believe that participation in the research study will not adversely affect the health of the patients who serve as research subjects.

15. Appropriate compensation and treatment for subjects who are harmed as a result of participating in research must be ensured.

Risks, Burdens and Benefits

16. In medical practice and in medical research, most interventions involve risks and burdens.

    Medical research involving human subjects may only be conducted if the importance of the objective outweighs the risks and burdens to the research subjects.

17. All medical research involving human subjects must be preceded by careful assessment of predictable risks and burdens to the individuals and groups involved in the research in comparison with foreseeable benefits to them and to other individuals or groups affected by the condition under investigation.

    Measures to minimise the risks must be implemented. The risks must be continuously monitored, assessed and documented by the researcher.

18. Physicians may not be involved in a research study involving human subjects unless they are confident that the risks have been adequately assessed and can be satisfactorily managed.

    When the risks are found to outweigh the potential benefits or when there is conclusive proof of definitive outcomes, physicians must assess whether to continue, modify or immediately stop the study.

Vulnerable Groups and Individuals

19. Some groups and individuals are particularly vulnerable and may have an increased likelihood of being wronged or of incurring additional harm.

    All vulnerable groups and individuals should receive specifically considered protection.

20. Medical research with a vulnerable group is only justified if the research is responsive to the health needs or priorities of this group and the research cannot be carried out in a non-vulnerable group. In addition, this group should stand to benefit from the knowledge, practices or interventions that result from the research.

Scientific Requirements and Research Protocols

21. Medical research involving human subjects must conform to generally accepted scientific principles, be based on a thorough knowledge of the scientific literature, other relevant sources of information, and adequate laboratory and, as appropriate, animal experimentation. The welfare of animals used for research must be respected.

22. The design and performance of each research study involving human subjects must be clearly described and justified in a research protocol.

    The protocol should contain a statement of the ethical considerations involved and should indicate how the principles in this Declaration have been addressed. The protocol should include information regarding funding, sponsors, institutional affiliations, potential conflicts of interest, incentives for subjects and information regarding provisions for treating and/or compensating subjects who are harmed as a consequence of participation in the research study.

    In clinical trials, the protocol must also describe appropriate arrangements for post-trial provisions.

Research Ethics Committees

23. The research protocol must be submitted for consideration, comment, guidance and approval to the concerned research ethics committee before the study begins. This committee must be transparent in its functioning, must be independent of the researcher, the sponsor and any other undue influence and must be duly qualified. It must take into consideration the laws and regulations of the country or countries in which the research is to be performed as well as applicable international norms and standards but these must not be allowed to reduce or eliminate any of the protections for research subjects set forth in this Declaration.

    The committee must have the right to monitor ongoing studies. The researcher must provide monitoring information to the committee, especially information about any serious adverse events. No amendment to the protocol may be made without consideration and approval by the committee. After the end of the study, the researchers must submit a final report to the committee containing a summary of the study's findings and conclusions.

Privacy and Confidentiality

24. Every precaution must be taken to protect the privacy of research subjects and the confidentiality of their personal information.

Informed Consent

25. Participation by individuals capable of giving informed consent as subjects in medical research must be voluntary. Although it

Copyright 2013 American Medical Association. All rights reserved.

Downloaded From: http://jama.jamanetwork.com/ by a American Medical Association User on 09/23/2015

may be appropriate to consult family members or community leaders, no individual capable of giving informed consent may be enrolled in a research study unless he or she freely agrees.

26. In medical research involving human subjects capable of giving informed consent, each potential subject must be adequately informed of the aims, methods, sources of funding, any possible conflicts of interest, institutional affiliations of the researcher, the anticipated benefits and potential risks of the study and the discomfort it may entail, post-study provisions and any other relevant aspects of the study. The potential subject must be informed of the right to refuse to participate in the study or to withdraw consent to participate at any time without reprisal. Special attention should be given to the specific information needs of individual potential subjects as well as to the methods used to deliver the information.

    After ensuring that the potential subject has understood the information, the physician or another appropriately qualified individual must then seek the potential subject's freely-given informed consent, preferably in writing. If the consent cannot be expressed in writing, the non-written consent must be formally documented and witnessed.

    All medical research subjects should be given the option of being informed about the general outcome and results of the study.

27. When seeking informed consent for participation in a research study the physician must be particularly cautious if the potential subject is in a dependent relationship with the physician or may consent under duress. In such situations the informed consent must be sought by an appropriately qualified individual who is completely independent of this relationship.

28. For a potential research subject who is incapable of giving informed consent, the physician must seek informed consent from the legally authorised representative. These individuals must not be included in a research study that has no likelihood of benefit for them unless it is intended to promote the health of the group represented by the potential subject, the research cannot instead be performed with persons capable of providing informed consent, and the research entails only minimal risk and minimal burden.

29. When a potential research subject who is deemed incapable of giving informed consent is able to give assent to decisions about participation in research, the physician must seek that assent in addition to the consent of the legally authorised representative. The potential subject's dissent should be respected.

30. Research involving subjects who are physically or mentally incapable of giving consent, for example, unconscious patients, may be done only if the physical or mental condition that prevents giving informed consent is a necessary characteristic of the research group. In such circumstances the physician must seek informed consent from the legally authorised representative. If no such representative is available and if the research cannot be delayed, the study may proceed without informed consent provided that the specific reasons for involving subjects with a condition that renders them unable to give informed consent have been stated in the research protocol and the study has been approved by a research ethics committee. Consent to remain in the research must be obtained as soon as possible from the subject or a legally authorised representative.

31. The physician must fully inform the patient which aspects of their care are related to the research. The refusal of a patient to participate in a study or the patient's decision to withdraw from the study must never adversely affect the patient-physician relationship.

32. For medical research using identifiable human material or data, such as research on material or data contained in biobanks or similar repositories, physicians must seek informed consent for its collection, storage and/or reuse. There may be exceptional situations where consent would be impossible or impracticable to obtain for such research. In such situations the research may be done only after consideration and approval of a research ethics committee.

Use of Placebo

33. The benefits, risks, burdens and effectiveness of a new intervention must be tested against those of the best proven intervention(s), except in the following circumstances:

    Where no proven intervention exists, the use of placebo, or no intervention, is acceptable; or

    Where for compelling and scientifically sound methodological reasons the use of any intervention less effective than the best proven one, the use of placebo, or no intervention is necessary to determine the efficacy or safety of an intervention

    and the patients who receive any intervention less effective than the best proven one, placebo, or no intervention will not be subject to additional risks of serious or irreversible harm as a result of not receiving the best proven intervention.

    Extreme care must be taken to avoid abuse of this option.

Post-Trial Provisions

34. In advance of a clinical trial, sponsors, researchers and host country governments should make provisions for post-trial access for all participants who still need an intervention identified as beneficial in the trial. This information must also be disclosed to participants during the informed consent process.

Research Registration and Publication and Dissemination of Results

35. Every research study involving human subjects must be registered in a publicly accessible database before recruitment of the first subject.

Copyright 2013 American Medical Association. All rights reserved.

Downloaded From: http://jama.jamanetwork.com/ by a American Medical Association User on 09/23/2015

36. Researchers, authors, sponsors, editors and publishers all have ethical obligations with regard to the publication and dissemination of the results of research. Researchers have a duty to make publicly available the results of their research on human subjects and are accountable for the completeness and accuracy of their reports. All parties should adhere to accepted guidelines for ethical reporting. Negative and inconclusive as well as positive results must be published or otherwise made publicly available. Sources of funding, institutional affiliations and conflicts of interest must be declared in the publication. Reports of research not in accordance with the principles of this Declaration should not be accepted for publication.

Unproven Interventions in Clinical Practice

37. In the treatment of an individual patient, where proven interventions do not exist or other known interventions have been ineffective, the physician, after seeking expert advice, with informed consent from the patient or a legally authorised representative, may use an unproven intervention if in the physician's judgement it offers hope of saving life, re-establishing health or alleviating suffering. This intervention should subsequently be made the object of research, designed to evaluate its safety and efficacy. In all cases, new information must be recorded and, where appropriate, made publicly available.

ARTICLE INFORMATION

Corresponding Author: World Medical Association, 13, ch. du Levant, CIB - Bâtiment A, 01210 Ferney-Voltaire, France; wma@wma.net.

Published Online: October 19, 2013. doi:10.1001/jama.2013.281053.

Disclaimer: ©2013 World Medical Association, Inc. All Rights Reserved. All intellectual property rights in the Declaration of Helsinki are vested in the World Medical Association. The WMA has granted *JAMA* exclusive rights to publish the English-language version of the Declaration through December 31, 2013.

Online-Only Content: Audio podcast is available at www.jama.com.

Copyright 2013 American Medical Association. All rights reserved.

Downloaded From: http://jama.jamanetwork.com/ by a American Medical Association User on 09/23/2015
